# MVS-Pheno: A Portable and Low-Cost Phenotyping Platform for Maize Shoots Using Multiview Stereo 3D Reconstruction

**DOI:** 10.34133/2020/1848437

**Published:** 2020-03-12

**Authors:** Sheng Wu, Weiliang Wen, Yongjian Wang, Jiangchuan Fan, Chuanyu Wang, Wenbo Gou, Xinyu Guo

**Affiliations:** ^1^Beijing Research Center for Information Technology in Agriculture, Beijing 100097, China; ^2^National Engineering Research Center for Information Technology in Agriculture, Beijing 100097, China; ^3^Beijing Key Lab of Digital Plant, Beijing 100097, China

## Abstract

Plant phenotyping technologies play important roles in plant research and agriculture. Detailed phenotypes of individual plants can guide the optimization of shoot architecture for plant breeding and are useful to analyze the morphological differences in response to environments for crop cultivation. Accordingly, high-throughput phenotyping technologies for individual plants grown in field conditions are urgently needed, and MVS-Pheno, a portable and low-cost phenotyping platform for individual plants, was developed. The platform is composed of four major components: a semiautomatic multiview stereo (MVS) image acquisition device, a data acquisition console, data processing and phenotype extraction software for maize shoots, and a data management system. The platform's device is detachable and adjustable according to the size of the target shoot. Image sequences for each maize shoot can be captured within 60-120 seconds, yielding 3D point clouds of shoots are reconstructed using MVS-based commercial software, and the phenotypic traits at the organ and individual plant levels are then extracted by the software. The correlation coefficient (*R*^2^) between the extracted and manually measured plant height, leaf width, and leaf area values are 0.99, 0.87, and 0.93, respectively. A data management system has also been developed to store and manage the acquired raw data, reconstructed point clouds, agronomic information, and resulting phenotypic traits. The platform offers an optional solution for high-throughput phenotyping of field-grown plants, which is especially useful for large populations or experiments across many different ecological regions.

## 1. Introduction

Plant genotyping and phenotyping technologies have significantly accelerated breeding programs focused on feeding the several billion people worldwide [1]. However, compared with the rapid development of genotyping technologies, the inability to efficiently and accurately capture complex phenotypic traits has become a bottleneck that limits progress in breeding programs [2, 3]. Substantial changes and improvements in phenotyping technologies for crops are required in the long term [4, 5]. Plant shoot architecture is one of the most important collections of phenotypic traits necessary for unleashing the full potential of plant science research [6]. Capturing these morphological traits provides a feasible way to assess the growth, physiology, stress responses, yields, and every developmental aspect of plants [7]. Such approaches are also fundamental to improving plant characterization, selection, and identification [8]. Therefore, developing accurate and efficient morphological data and processing approaches is of great significance for plant phenotyping and further plant breeding efforts.

Early phenotyping technologies are usually time consuming, low throughput, and labor intensive. Thus, many phenotyping platforms have been developed for the purpose of throughput and efficiency improvements [9–11]. According to their working context, existing platforms can be categorized into field and indoor phenotyping platforms.

Field phenotyping platforms mainly consist of unmanned aerial vehicle (UAV) remote-sensing platforms [12], cable-suspended field phenotyping platforms [13], robotic field phenotyping platforms mounted on fixed rails [10], and tractor-driven field phenotyping systems [14, 15]. Field phenotyping platforms are integrated with moveable sensors on robotic carriers that enable them to acquire morphological and physiological data from crops and are thus described as “sensor-to-plant” systems [16]. These platforms have been used to evaluate the field performance and adaptability of crops in natural field conditions. Plot- or canopy-scale phenotyping parameters, such as plant height, leaf area index (LAI), canopy cover, and above-ground biomass, can be efficiently estimated using these platforms [17]. To achieve data acquisition efficiency for large-scale canopies, the resolution of sensors on these platforms is often relatively low. Furthermore, the occlusion of adjacent plants tends to obstruct the accurate capture of finer phenotypic traits by field phenotyping platforms.

Precise phenotypic traits are of value for genome-wide association studies (GWAS) and enabling advances in crop breeding [18, 19]. Thus, indoor platforms have been developed to obtain more fine-scale data on phenotypic traits. Indoor platforms include conveyor-based platforms for phenotyping individual pot-grown plants [20, 21], robot-assisted imaging pipelines for tracking the growth of individual plants [22], chamber monitoring systems for small plants [23], and microscale phenotyping for interior structure analysis [24]. Most indoor phenotyping platforms have fixed sensors, and therefore, plant samples are transported to the imaging system, which is thus described as “plant-to-sensor” systems [16]. Accordingly, high-resolution sensors can be used to acquire morphological and physiological data, and detailed phenotypic traits, such as leaf length, leaf area, leaf angle, and growth rate, can be derived at a large scale.

Most of the above phenotyping platforms are expensive to build, operate, and maintain. This prevents many researchers from implementing these urgently needed approaches owing to their insufficient budgets. Therefore, affordable phenotyping solutions [25] are needed. Common sensor choices for acquiring morphological data to achieve affordable phenotyping include RGB cameras [26], two-dimensional (2D) LiDAR [27], and three-dimensional (3D) scanners [28]. Accuracy, efficiency, and cost are three key attributes of these sensors.

Maize (*Zea mays*) is one of the most widely grown crops worldwide. It is predicted that more than half of the increased food demand for cereal plants will come from maize [19]. Accordingly, substantial changes in phenotyping technologies for breeding and crop improvement will be required [29]. As precision phenotyping of individual plants will benefit GWAS and crop breeding research, many researchers have developed high-throughput and efficient phenotyping platforms and methods to capture maize plant traits. Phenotypic parameters derived from 2D images taken from appropriate angles [22] are satisfactory for many research demands. However, these data often lack depth information and require some extracted parameters to be calibrated, such as leaf azimuthal angle, leaf length, and leaf area. As such, 3D reconstruction of plants is an alternative way to solve this problem. Commonly adopted 3D reconstruction approaches include 2D LiDAR synthesis [27], time-of-flight camera reconstruction [30, 31], multiview stereo (MVS) reconstruction [32–34], 3D digitization [35], and 3D laser scanning [36].

MVS reconstruction has been demonstrated to be more efficient than 3D scanning and digitization, and its low cost and one-by-one pipeline data acquisition pattern make it a better choice among affordable and portable field phenotyping platforms [35]. However, MVS image sequences are acquired manually in most plant phenotyping applications, which is time consuming and labor intensive. Manually capturing MVS images seems infeasible for GWAS, as recombinant inbred line populations contain many recombinant individuals [29]. Moreover, neighboring images acquired by manually operations might not be satisfactory for MVS reconstruction, and this data deficiency is often discovered while using postprocessing applications, which is too late. PlantScan Lite, an automatic MVS-based phenotyping platform with dual cameras, was built according to the plant-to-sensor pattern [37]. However, it requires about 30 minutes to obtain all MVS images of an individual plant, which is ineffective and unsuitable for high-throughput phenotyping. Additionally, a low-cost 3D imaging system was built for quantifying variation in soybean crops caused by flooding [38]; an arm-mounted camera in the system allows the capture of images along a defined trajectory of a circle in a plane during automatic MVS image acquisition. However, owing to the limited circle range, the system is only suitable for small crops.

Plants grown in controllable environments are not representative of crops under natural field conditions, especially when evaluating the adaptability and resistance of new cultivars under certain cultivation strategies. Though indoor phenotyping platforms are capable of acquiring fine-scale phenotypes of individual plants, high-throughput 3D phenotyping of field-grown maize still remains a practical problem. Besides, the plant height of maize shoots changes across a wide range dynamically throughout the entire growth period. Humans need to climb ladders around a shoot to acquire the full MVS images of tall shoots, which is laborious and time consuming. Accordingly, to create a high-throughput and fine-scale phenotyping solution for individual plants in fields of maize, we developed MVS-Pheno, a portable and low-cost phenotyping platform using MVS reconstruction. Automatic and high-efficiency MVS image acquisition of maize shoots can be conducted using the platform. Additionally, a data acquisition console, phenotype extraction software, and data management systems were also developed specifically to facilitate use of the platform.

## 2. Methods

### 2.1. Overview of the Platform

**Figure 1:**
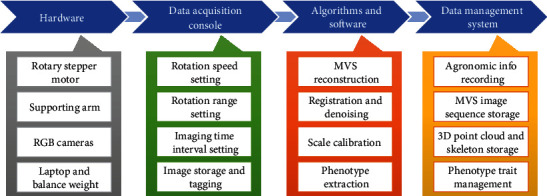
Components of the MVS-Pheno platform.

The MVS-Pheno platform includes four components (Figure 1). (1) The hardware for automatic MVS image acquisition is composed of a rotary stepper motor for rotating the arm and cameras, a supporting arm for holding the cameras, RGB cameras to acquire the MVS images, a laptop for data storage and hardware control, and a balance weight to ensure system stability while rotating. (2) The data acquisition console is a module setup on the laptop that is used to interactively handle the hardware settings for MVS data acquisition. The setting parameters include rotation speed and range, imaging time interval, and image storage path and tagging. (3) The algorithms and software have been developed specifically for maize and include integrated modules for MVS reconstruction, point cloud registration and denoising, scale calibration, and phenotype extraction. (4) The data management system consists of a database built to organize and manage the data flow using the MVS-Pheno platform. The input agronomic information, the original acquired MVS sequences, the reconstructed 3D point clouds and extracted skeletons, and the phenotypic traits of corresponding individual plants are organized in the database.

### 2.2. Design of the MVS Image Acquisition Device

**Figure 2:**
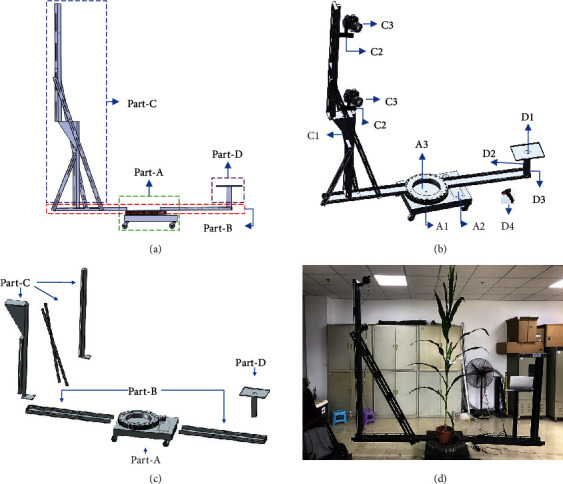
The complete device, including the lateral view (a), a stereogram (b), breakdown drawing (c), and a scenario in which the device is being used for data acquisition (d).

An MVS image acquisition device was designed for the following aims: (1) to perform automatic MVS image acquisition under uniform standards, (2) to offer an easy to disassemble and assemble device with portability and usability in any agricultural field, and (3) to improve image acquisition efficiency using two or more cameras simultaneously. The device designed with these objectives in mind is illustrated in Figure 2.

#### 2.2.1. Structure of the Device

The device is composed of four parts, as illustrated in Figure 2, consisting of part-A to part-D. (1) Part-A is the driving component of the device. A1 is a circular gear bearing, with a built-in rotary stepper motor that drives the rotation of the arms and cameras with a uniform velocity. A2 is a rotation controller that controls the rotation angle of the rotary machine. A3 is the support table, which has four wheels at the bottom to facilitate device movement. Plant samples are placed in the center of the table. (2) Part-B is composed of two beams that connect Part-A to Part-C and Part-D. The length of each beam is adjustable according to the height of the target shoot. (3) Part-C is the vertical arm. It is composed of a supporting arm (C1), several camera mounts (C2), and multiple cameras (C3). The length of the supporting arm and the number of cameras used are also adjustable to ensure the full overlap and consistency of MVS image sequences and also suit a range of shoot heights. The length adjustment of both beams in part-B and supporting arms in part-C is realized by inserting small extensions. (4) Part-D is the balance weight (D3) of the device, which is attached to the other beam of the device. A laptop can be placed on the supporting table (D1), and a portable power source (D2) can also be placed on part-D to provide sufficient power to the laptop for lengthy data acquisition periods. In addition, a wireless bar code scanner (D4) is included to enable the rapid capture of agronomic information about the target shoot.

The device is composed of these basic components, which makes the device easy to reproduce, assemble, and transport.  Figure 2(c) presents the breakdown drawing of the device. The device can be assembled by simply connecting these components using bolts.  Table 1 shows the component costs of the device, which is $7560 in total for a standard device with two camera sensors. Compared with the expensive infrastructure and sensors typically required for plant phenotyping, this platform is relatively low-cost.  Table 2 presents the weight and minimum length parameters of the main components of the device, which determine the portability and transportation cost of the device. The shortest length of all the components is 120 cm, and the total weight of all the components is around 140 kg. Thus, the device can be transported by logistics or express delivery over long distances for experiments or by a minivan for nearby experiments. A collection of “sldprt” and “sldasm” format files of the components and the device can be found in Supplementary Materials (available here), with detailed dimensional drawings and assembly procedures.

In addition, four LED white light sources should be arranged around the device to ensure brightness and light uniformity, especially when the light conditions of the experimental site are not satisfactory.

#### 2.2.2. Technical Parameters of the Device

The device is assembled to match the target shoot height and size of the space to be used to conduct the data acquisition. The three primary technical parameters of the device are defined as follows:

*N*: the number of cameras used simultaneously by the device

*R*: the radius (in m) of both of the beams, including half of the circular gear bearing

*H*: the height (in m) of the supporting arm

The size of the device for a maize shoot with a height of *h* can be described as *V*_*h*_ = {*H*, *R*, *N*}. For a maize shoot before the V6 period [39], with a height of less than 0.6 m, the device can be adjusted to *V*_0.6_ = {1.0, 0.8, 1}. For a maize shoot at the silking stage, with a height of about 2.0 m, the device should be adjusted to *V*_2.0_ = {2.5, 1.5, 3} to ensure the overlap of the captured MVS images. Maize may grow up to 4.0 m in some ecoregions with adequate sunlight and temperatures [40]. In these cases, a larger field of view is needed. Thus, the device can empirically be adjusted to larger than the 5.0 m height of the supporting arm and the 2.0 m length of each beam. However, this would significantly increase the weight and transportation costs of using the device. Therefore, tall shoots can be segmented into two parts to acquire the images and register the point clouds.

Canon 77D cameras (Canon Inc., Tokyo, Japan) are used as the image acquisition sensors in the standard device. Their focal lengths are 24 mm, and the resolutions are 24 million pixels. The cameras that can be used are not limited to this specific model. The cameras are aimed downward towards the vertical center line of plant shoots, forming an angle of around 45° with the horizontal plane. Each camera is responsible for acquiring a layer of images. Images captured by adjacent cameras must overlap by at least 1/3.

### 2.3. Data Acquisition Console

A console was developed to connect the cameras, laptop, and rotary stepper motor in order for them to work together and enable automatic MVS image acquisition.

#### 2.3.1. Data Acquisition Process Using the Device

**Figure 3:**
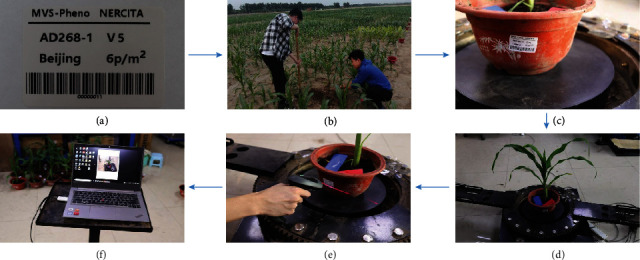
Data acquisition workflow using MVS-Pheno. Preparing labels for a shoot in an experiment (a), including the bar code, cultivar name (AD268), growth stage (V5), ecoregion (Beijing), and planting density (6 plants/m^2^). Transplanting shoots to pots in the field (b). The prepared bar code affixed to its corresponding pot (c). The pots and shoots placed on the device (d). The device is started by scanning the bar code using a wireless bar code scanner (e). Automatic image sequence acquisition using the console (f).

Bar codes along with text information can be prepared for each shoot used in the experiment. Bar code information typically includes the cultivar name, density, growth period, ecoregion, and shoot ID (Figure 3(a)). First, a room or a temporary tent that can accommodate the device has to be found or built at the data acquisition site near the experimental field. Then, the device is assembled in the chosen space. Target maize shoots are transplanted into pots carefully, along with the soil beneath them to a depth of 10–30 cm, depending on the size of the rhizosphere (Figure 3(b)). The pots used are of the same size. The prepared bar codes for the shoots are posted on the corresponding pots (Figure 3(c)). These shoots and pots are transported to the device carefully to minimize damage to the morphology of the plants. The shoots with pots are placed on the device immediately to perform the MVS image acquisition, one-by-one (Figure 3(d)). For the whole data acquisition procedure, the first half of the steps are performed manually and the latter half of the data acquisition process is conducted using the device. Thus, the whole data acquisition process is semiautomatic.

#### 2.3.2. Control Signal and Data Transmission

The console runs on the laptop, which is connected to the rotation controller (A2 in Figure 2) via a wireless communication module. The console sends user-specified rotation parameters to the rotation controller, including the total angle range (*φ*), the velocity (*v*) of the rotation, and the moment to start up the rotation. Micro USB-B data transmission cables, which are the supporting data line of the cameras, are used to connect the cameras to the laptop. The ID of the cameras in the device are identified. Thus, the console is able to send signals to all the cameras synchronously, and the acquired images are received from each camera through the cables, respectively. Each group of the acquired images for a shoot sample is saved to a file directory and tagged with the sample name based on the user input via the console interface. The wireless bar code scanner (D4 in Figure 2(b)) can be used as an alternative and convenient way to start up and input the agronomic information (Figure 3(e)). The time interval (*t*) for capturing images is also specified by the user through the console interface. Owing to the exposure time for the cameras, the time interval has to exceed 1 s. The number of images in each layer (*n*) can thus be calculated.

#### 2.3.3. Common Settings

Most user-specified parameters input via the console are empirical variables for maize shoots, including the total angle of rotation range, the velocity of the rotation, and the image capture time interval of the camera. These parameters are fixed for most sets of samples. Users only need to specify the sample name and press the start button on the console. To ensure the stability of the rotatory motor, *v* is limited to lower than 1 minute per circle (6°/s). The rotation range of a circle is set to 400° to ensure a satisfactory overlap between the first and last images captured.  Table 3 shows the empirical settings in our experiments with maize shoots.

### 2.4. Point Cloud Processing and Phenotype Estimation

#### 2.4.1. Overview of the Point Cloud Processing Pipeline

**Figure 4:**
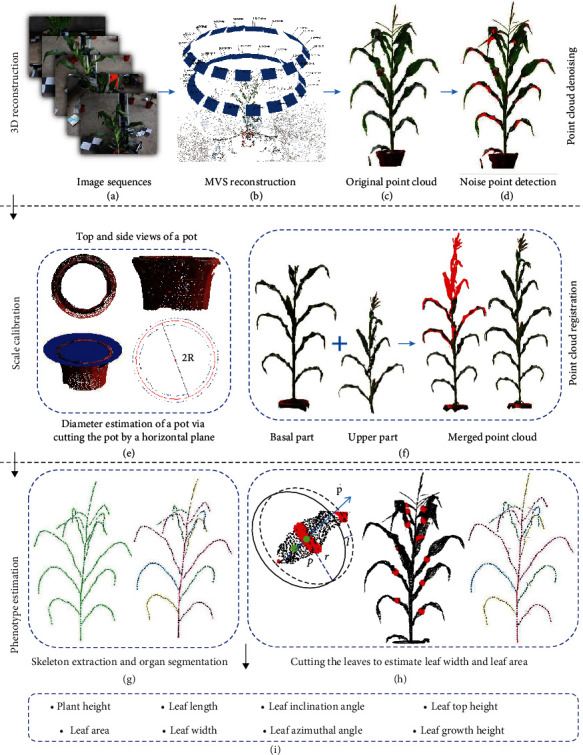
Illustration of point cloud processing pipeline.

The acquired data, including the image sequences and corresponding information, are copied to a workstation. A series of operations are conducted on the images to reconstruct and extract phenotypes of the individual plants. First, 3D point clouds are derived using MVS reconstruction by inputting the acquired image sequences. Then, noise in the point cloud of a shoot is detected and removed. After that, the shoot point cloud is calibrated to its real size by calculating the size of the pot in the point cloud, whose actual size is known already. Shoots that are too tall to be acquired by the device can be cut into two segments to enable the MVS images to be acquired separately, and the point clouds of the two segments can then be registered to obtain the complete point cloud. At last, skeletonization and leaf segment sampling are conducted on the point cloud of maize shoots. Based on these data, phenotypic traits relevant to shoot architecture are finally derived.  Figure 4 illustrates the point cloud processing pipeline.

#### 2.4.2. MVS Reconstruction

The commercial software Agisoft Photoscan Professional (Agisoft LLC, St. Petersburg, Russia), which performs photogrammetric processing of digital images, is used to recover dense point clouds with colors from the acquired MVS images (Figures 4(a)–4(c)). This software is commonly used in MVS-based plant phenotyping [41, 42]. Photoscan is able to reconstruct 3D objects automatically using a series of ordered or unordered images with overlapping areas. The moving trajectory and coordinates of the camera are not needed during the processing.

#### 2.4.3. Denoising of Point Clouds

The reconstructed point clouds of individual plants often contain lots of noise from the backs of leaf blades, forming areas with insufficient data owing to the uneven illumination and camera angles of views (Figure 4(c)). This noise affects the accuracy of generated 3D mesh models and thus the extracted phenotypic traits. Notably, there are significant color differences between the leaves and noise associated with the backs of leaf blades, and the color of the noise is continuous. Therefore, a region growth denoising algorithm [43], constrained by color differences, was applied. This algorithm utilizes a low-cost approximation color metric model (https://www.compuphase.com/cmetric.htm) to improve the efficiency of denoising. The model is shown as follows:

(1) $$\begin{array}{c} \bar{r}=\frac{R_{i}+R_{j}}{2},\\ \Delta R_{ij}=R_{i}-R_{j},\\ \Delta G_{ij}=G_{i}-G_{j},\\ \Delta B_{ij}=B_{i}-B_{j},\\ \Delta C_{ij}=\sqrt{\left(2+\frac{\bar{r}}{256}\right)\times \Delta R_{ij}^{2}+4\times \Delta G_{ij}^{2}+\left(2+\frac{255-\bar{r}}{256}\right)\times \Delta B_{ij}^{2}}. \end{array}$$

Here, *R*_*i*_, *G*_*i*_, and *B*_*i*_ are the red, green, and blue values of the *i*th point and Δ*C*_*ij*_ is the color difference between the *i*th and *j*th points.

In the region growth denoising algorithm, a noise point color list *C*_*L*_ is constructed. The list is a priori data list that is generated by noise sampled from different illumination experiments. As mentioned, most noise is associated with the backs of leaves, which appear gray in the absence of light. Thus, the list is constructed by manually collecting the colors of noises under different light conditions across different leaf positions. A point cloud list *P*_*L*_, composed of a few points with approximate colors of the elements from list *C*_*L*_, is generated by simply sampling from point cloud set *P*. For each point *p* ∈ *P*_*L*_, the color difference Δ*C*_*pq*_ and normal angle *θ*_*pq*_ between point *p* and all the points *q* belonging to the *k*-neighborhood of *p* are calculated. Finally, a discriminant threshold *δ*_*pq*_ is derived for the noise point classification:

(2) $$\begin{array}{c} \delta_{pq}=\eta_{1}\Delta C_{pq}+\eta_{2}\theta_{pq}, \end{array}$$

where *η*_1_ and *η*_2_ are the weight coefficients of Δ*C* and *θ*, respectively. As a larger of Δ*C* or smaller *θ* indicates that the point may be noise, we empirically set *η*_1_ = ‐1/40, and *η*_2_ = 1/90. The normal angle *θ* is used to estimate the normal consistency of the two points to avoid false deletion of points lying on the leaf margin. If *δ*_*pq*_ is smaller than an empirical threshold, point *q* is identified as a noise point and is pushed into list *P*_*L*_. After all the points belonging to the *k*-neighborhood of *p* are estimated, the seed point *p* is removed. Then, all the points in *P*_*L*_ are traversed until it is empty and the denoising process is complete. The visualization results are shown in Figure 4(d).

#### 2.4.4. Scale Calibration

Point clouds reconstructed using MVS algorithms or software are scaled [33]. Thus, the point cloud model has to be transformed to its actual size, especially for the eventual phenotype estimation. Scaling markers, with regular and relatively complete shapes, have to be found and identified. First, the visibility of the pots containing the plants is ensured, and thus, complete point clouds of them can be obtained. Second, it is easy to segment the point clouds of pots by identifying their uniformly distributed color. Therefore, pots are selected as markers for correcting the size of reconstructed shoots in the MVS-Pheno platform. The segmented point clouds are illustrated in Figure 4(e). A set of circular ring point clouds is derived by manually cutting the pot along a horizontal plane. Then, the radius of the pot is derived by using the least squares approach to fit the points along the cuts. Finally, the metric scale factor between the estimated radius from the point cloud to the actual radius of the pot is considered as the corrective scale for the shoot. The estimation is depicted in equation (3), where *τ* is the estimated scale factor, *R* is the measured radius of the actual pot, and *R*_MVS_ is the estimated radius of the pot from the point cloud:

(3) $$\begin{array}{c} \tau =\frac{R}{R_{\text{MVS}}}. \end{array}$$

To reduce the scale error, three slices are used for each pot in practice, including the top, middle, and bottom of the pot. Then, the average value at the three estimated positions is used as the final scale factor. The point cloud coordinates obtained from the MVS-Pheno platform are multiplied with the scale factor to reach a scaling in millimeters.

#### 2.4.5. Point Cloud Registration

Owing to the height limitation of the device, upper leaves of some tall shoots can be out of the imaging range of the device. Accordingly, it is infeasible to acquire full MVS images for these shoots. For such shoots, we first acquire the basal images of each shoot using the device. Then, the shoot is segmented manually using branch scissors, ensuring that the upper parts after segmentation have at least two complete leaves to be validated in the first image acquisition process. Then, the upper portion images are acquired once again using the device. Point clouds are then derived using MVS reconstruction of the basal and upper parts of a shoot. Leaves repeated in the two point clouds are selected, and the interactive closest point (ICP) algorithm [44] is used to register these two point clouds. In the ICP algorithm, a rotation matrix *R* and translation matrix *T* are estimated to minimize *E*(*R*, *T*), as shown in equation (4). Using the estimated matrix, the two point clouds are well registered. The reconstructed point cloud of tall shoots using truncation and the registration approach is shown in Figure 4(f) by the following:

(4) $$\begin{array}{c} E\left(R,T\right)=\frac{1}{N_{p}}\overset{N_{p}}{\underset{i=1}{\sum }}{\left\|p_{i}-{Rq}_{i}-T\right\|}^{2}. \end{array}$$

In equation (4), *R* and *T* are the rotation and transmission matrices to be estimated, *P* and *Q* are two point clouds to be registered, and *p*_*i*_ ∈ *P*, *q*_*i*_ ∈ *Q*. *N*_*P*_ is the total point number of *P*.

#### 2.4.6. Phenotype Extraction of Individual Plants by Skeletonization

The 3D skeleton of maize shoots exhibit important maize phenotypes. In our previous study [45], an accurate approach that extracted phenotypes from 3D point clouds was proposed to obtain the skeleton and phenotypic traits of maize shoots. In this approach, the Laplacian contraction algorithm was applied to shrink the initial skeleton points (Figure 4(g)). Then, deviation skeleton points of maize stems and leaves to the input point cloud were calibrated by building a step forward local coordinate along the tangent direction of the original points. Finally, six phenotypes, including plant height, leaf length, leaf inclination angle, leaf top height, leaf azimuthal angle, and leaf growth height, can be captured using the extracted skeleton with high accuracy. Based on these data, we aim to obtain leaf width and leaf area traits. First, cubic spline interpolation is conducted by inputting the skeleton points of each leaf. Then, uniformly distributed points on the skeleton curve are derived by calculating the output of the cubic spline through inputting equidistance parameters. Thus, points on the skeletons of leaves are uniformly distributed and smoothed. After that, five to eight nodes evenly distributed on the extracted skeleton are selected, forming a set of tangent points of each leaf. For each node *p*, the corresponding tangent $\vec{p}$ is then evaluated based on the skeleton points (Figure 4(h)). A set of point clouds is selected by cutting a virtual ring perpendicular to $\vec{p}$ on the reconstructed point cloud of the shoot; the center of the ring is *p*. The radius *r* of the ring is set to 1.5 times the average leaf width (manually measured), and the length *l* is twice the average distance of the adjacent points of the point cloud. Overcutting points were selected and deleted using the nearest neighbor clustering method [46], and the point set with the most clustered points is considered as the final point cloud. Finally, the cutting line segment is fitted using a least squares approach. The longest value of the cutting line segment is taken as the leaf width. The areas of the continuous and segmented trapezoids are calculated and summed as the area of each leaf. At last, eight phenotypic traits can be obtained as presented in Figure 4(i).

#### 2.4.7. Software Development of MVS-Pheno

Data processing software was developed by integrating the aforementioned algorithms and implemented using the Point Cloud Library (PCL) on the Microsoft Visual C++2010 platform. The software is compatible with the Windows operating system (Windows 7 and above) and requires more than 8 GB of memory and a processor faster than 3.2 GHz. The software includes four functional modules: (1) an MVS image acquisition module for data acquisition and management; (2) a 3D visualization module for data display and interaction; (3) a data processing module for point cloud processing, skeleton extraction, and phenotypic trait calculation; and (4) a pipeline recording module for logging the operations of computational processes.

### 2.5. Data Management System

**Figure 5:**
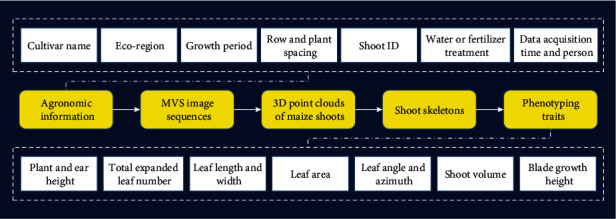
Data flow and management in the MVS-Pheno platform database.

Phenotyping platforms generally acquire a large number of biological datasets, most of which cannot be processed into agronomic phenotypic traits immediately; thus, phenotyping databases [47] or information management systems [48, 49] are required to support data management. Huge amounts of data can be generated using the MVS-Pheno platform (Figure 5), including agronomic information about the designed experiments, MVS image sequences acquired during the device use, reconstructed 3D point clouds of maize shoots, extracted shoot skeletons, and phenotypic traits derived using the software. Therefore, a data management system has to be established for MVS-Pheno to ensure orderly data storage, further processing, and management. In practice, a customized database can be established for any given experiment. Data that require large storage space, including MVS images, reconstructed point clouds, and shoot skeletons, are stored in file directories, organized by agronomic information within subdirectories. Agronomic information, phenotypic traits, and the corresponding file paths are stored in the database. Consequently, different data from each procedure could be connected and traced using the database.

## 3. Materials

A field experiment was conducted utilizing the MVS-Pheno platform in 2018 at an experimental field at the Beijing Academy of Agriculture and Forestry Sciences (39°56′N, 116°16′E). A maize hybrid AiDan268 (AD268) was planted in three replicate plots on June 4, 2018. The planting density was 6 plants/m^2^, with a row spacing of 60 cm. At the V5, V15, and R1 growth stages [39], one shoot was selected from each plot (i.e., three shoots for each growth stage in total) and transplanted with their root systems and associated soil into 30 cm diameter, 25 cm deep pots containing soil [35].  Table 4 summarizes the selected shoots. The shoots with pots were moved indoors, where 3D data were obtained. First, image sequences were acquired using the MVS-Pheno platform. Then, a 3D scanner (FARO^3D^ X120; FARO, Lake Mary, FL, USA) was used to acquire 3D point clouds for evaluation of 3D models. Finally, a 3D digitizer Fastrak (Polhemus, Colchester, VT, USA) was used to acquire the 3D feature points of the shoots. As mentioned by Wang et al. [35], the promising accuracy of 3D digitizing provides a reasonable means of verifying 3D phenotyping approaches. Herein, phenotypic traits were extracted directly using the 3D digitized data for the accuracy evaluation of phenotypes derived with the MVS-Pheno platform.

## 4. Results

### 4.1. Evaluation of 3D Point Cloud Accuracy

**Figure 6:**
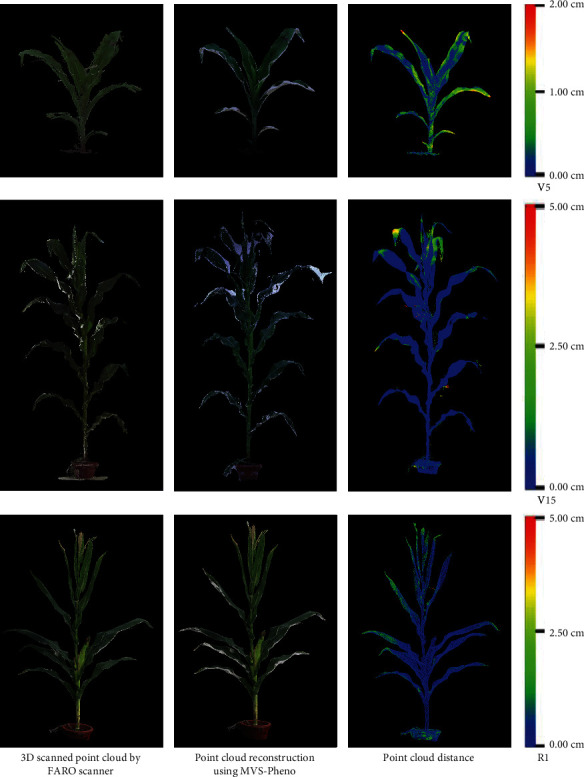
Comparison of point clouds of maize shoots at different growth stages derived using a 3D scanner and MVS reconstruction with images captured using the MVS-Pheno platform.

Point clouds were obtained using a 3D scanner to evaluate the reconstruction accuracy of maize shoots with the MVS-Pheno platform. Three shoots of the same hybrid (AD268) were selected from the center of the plot from each of the three replicates at three different growth stages. The shoots were scanned using the FARO scanner after MVS image acquisition. The reconstruction accuracy was evaluated by comparing the point clouds derived by the two approaches [35].  Figure 6 illustrates the visualization of the comparison. The point clouds reconstructed by MVS-Pheno match well with the scanned point clouds. Point cloud differences also existed only in some local areas of the organs. The maximum distances were 2.0 cm for V5 stage shoots and 5.0 cm for V15 and R1 shoots (Figure 6). In addition, the reconstructed point clouds of the V5 stage shoots present better morphological consistency with the real shoot, while the quality of the point clouds derived using the FARO scanner was relatively poor, with many outliers. The comparison demonstrates that point clouds of maize shoots reconstructed using MVS-Pheno are satisfactory for different growth stages, and better results can be obtained with it for small shoots relative to some 3D laser scanners.

### 4.2. Evaluation of Extracted Phenotypic Traits

**Figure 7:**
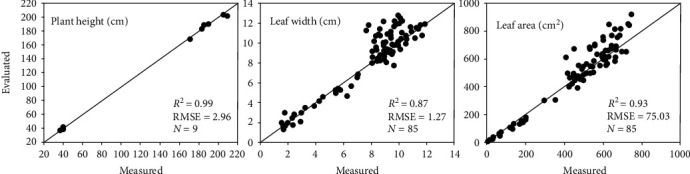
Comparison of plant height, leaf width, and leaf area derived using the phenotypic trait extraction algorithm in MVS-Pheno.

In our previous study, several phenotypic traits estimated from extracted skeletons were validated, including plant height, leaf length, leaf angle, and leaf azimuthal angle [45]. Here, we focus on leaf width and leaf area. Based on the scale calibration operation involved in point cloud reconstruction, plant height was also validated again to ensure that the scaling was correct. Additionally, plant height is measured as the distance between the natural bend of the uppermost leaves or tassel [39] to the soil surface in the pot after the shoot is rotated perpendicular to the horizontal plane.  Figure 7 shows the verification of the three phenotypes using the materials in Table 4. The plant height correlation (*R*^2^) between the measured and evaluated values was 0.99, which indicates that the scaling algorithm performs well for estimation. The correlation for leaf width was lower (*R*^2^ = 0.87), while the correlation for the leaf area was higher (*R*^2^ = 0.93). The correlations suggest that the present approach is acceptable for estimating leaf width and accurate for estimating the leaf area.

### 4.3. Efficiency Performance of MVS-Pheno

The data capture device of the platform has been used in many experiments at several ecological sites throughout China, including Qita farm in Xinjiang, Gongzhuling in Jilin, Sanya in Hainan, and Tongzhou district in Beijing. During the experiment, we summarized the time cost of each procedure and the corresponding parameter settings of the device for different growth stages (Table 5). Thus, plant height is the key determining index for the parameters and time costs. For plant taller than 250 cm, the shoot must be cut into two segments, and thus, the time for image acquisition is more than twice as long than that required for shorter plants. When the shoots are relatively short (V3 to V6 growth stages), the data acquisition and 3D reconstruction of three or four shoots can be conducted together to save time. Point cloud reconstruction using commercial software requires nearly 66% to 88% of the total time consumed. If the point cloud of a shoot is rather complex, manual intervention is required to correct errors in the process of skeleton extraction and phenotype estimation. The postprocessing operations were conducted on a desktop workstation (Intel core i7 processor, 3.2 GHz CPU, 64 GB of memory, Windows 10 operating system).

### 4.4. Data Management System Interface

**Figure 8:**
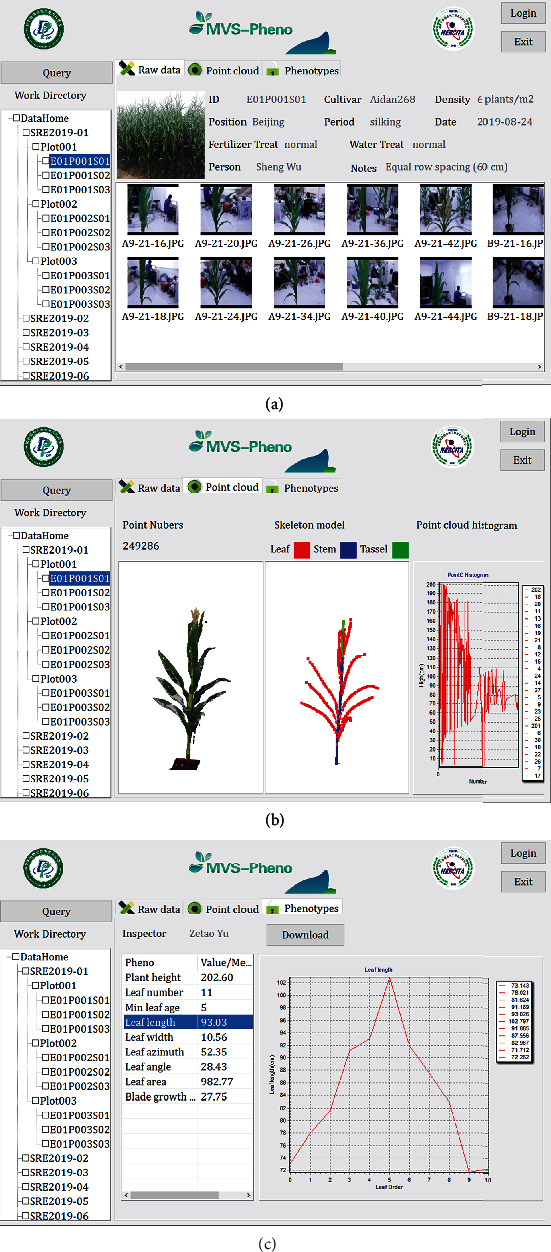
Interfaces of the data management system developed for the MVS-Pheno platform.

Data in the MVS-Pheno data management system is organized at three levels, and a tree directory guides the users in finding the data of interest. The first level is the experiment level. Users can add their experimental name as a subdirectory under the main directory, named DataHome (Figure 8). When this addition is conducted, a subdatabase is constructed and the acquired data have to be copied into this directory. Meanwhile, the corresponding database (constructed when acquiring the MVS images) has to be added into the database of the data management system. The second level is the plot level. Data in each plot are organized in an independent folder, and all the plot data are stored in the experimental folder. Individual plants are considered as the basic data unit in the data management system. Thus, the shoot samples in each plot are the third level of the system. In addition, when right clicking the DataHome button or the button for each experiment name, statistical information of the selected (sub-)database is shown to the user, including the total plot number, total shoot number, total storage, and data processing progress. Users can thus find the general information about the data and the data processing process by checking the statistical information.

When a shoot sample is selected in the directory or is searched for in the query dialog, the data is shown in the main screen of the interface. The data include three parts: raw data, point clouds, and phenotypes (Figure 8). The first part shows the raw image sequences and the corresponding information for the selected shoot (Figure 8(a)). Detailed information includes the shoot ID, cultivar name, density, position, growth period, data acquisition date, fertilizer and water treatments, person who acquired the data, and other notes. Raw images were named according to the camera ID plus the acquisition time. For example, an image with the file name “B9-21-16” was acquired at 9:21:16 by camera B. When the MVS reconstruction is conducted, the second part, the “Point cloud and skeleton” dialog, is activated. In this part, three views present the reconstructed point cloud, the point histogram along plant height, and the extracted skeleton of the shoot (Figure 8(b)). The third part presents a list of the extracted phenotypic traits and the statistical information about the selected shoot. Determined values are given directly in the list, such as plant height, total leaf number, and leaf age. For phenotypes that contain many values, such as leaf length, the average value of all the extracted leaf lengths is presented in the list. When selecting to view this averaged value, the detail parameters and the corresponding line chart will be presented in the right widget on this interface (Figure 8(c)). Finally, the phenotypes and point cloud distribution histogram can be output as an independent file for further analysis and applications.

## 5. Discussion

### 5.1. Field-Grown Shoot Phenotyping

Plant phenotyping aims at assessing the morphological and physiological performance of plants with various genotypes in different environments. As field environments are complex and changeable, and it is the actual environment that most plants experience, field-based phenotyping has been increasingly recognized as the only approach capable of delivering the requisite trait expression data for numbers of plants or populations in real-world cropping systems [50]. Thus, field phenotyping technologies are very important for plants. Though UAV remote sensing [12] is applicable for large-scale field phenotyping with high efficiency, the resolution is lower than ground phenotyping platforms and only canopy-scale traits can be obtained. Field phenotyping infrastructures [10, 13] are also capable of acquiring time series phenotypic data with higher resolution. However, plant organs grown at the middle and bottom of the canopy are sheltered from the upper organs, which prevents estimating fine-scale phenotypes of individual plants in the field. Meanwhile, these infrastructures are position limited with expensive construction costs. It is not feasible to obtain fine-scale phenotypes for breeding and cropping experiments with many plant populations across several ecoregions. Manned ground vehicles (MGVs) have become popular and provide reasonable solutions for field plant phenotyping. Semicontrolled crop-sensing platforms, such as tractor-based platforms [51], carry sensors within or above plants to acquire data. This eliminates the constraint of phenotyping position compared with infrastructures. Nevertheless, MGVs are also unable to extract the fine-scale phenotypes of individual plants. Most large-scale phenotyping studies at the individual plant level are conducted with pot-grown plants cultivated carefully in controlled environments [11, 29]. However, results from controlled environments are far removed from the situations plants experience in the field and are thus difficult to extrapolate to the field [52]. Therefore, high-throughput phenotyping solutions for individual field-grown plants are required. The portability of the MVS-Pheno platform enables phenotyping of field-grown maize shoots at any experimental site. Meanwhile, the device is low-cost and feasible for most institutes. Compared with the aforementioned approaches, the MVS-Pheno platform is a more cost-effective solution for fine-scale phenotyping of individual plants. However, this solution is destructive and only semiautomatic (i.e., plants must be transplanted into pots and moved to the platform manually); thus, in situ continuous monitoring of specific maize shoots is difficult, and manual transplants during the data acquisition process are needed.

### 5.2. Comparison with MVS-Based Phenotyping Platforms

MVS-based 3D reconstruction has become a popular approach for low-cost plant phenotyping. The MVS-Pheno platform offers an automatic way to acquire the image sequences of plant organs or shoots. Compared with manual imaging [32, 34], the most used approach to acquire MVS images, the MVS-Pheno platform is highly automated and liberates manpower in the data acquisition process, especially for tall shoots. Humans do not need to climb a ladder or hold a supporting pole with a camera to acquire the MVS images of tall shoots. Image acquisition for tall shoots is also a difficult problem for other low-cost MVS-based phenotyping platforms [37, 38, 53]. In addition, the data acquisition efficiency of the platform is another advantage over other similar platforms. For a normal size shoot, approximately 60 seconds are required to acquire all the necessary images using MVS-Pheno, while the PlantScan Lite platform [37] requires 30 minutes. This high data acquisition efficiency is quite important for plant phenotyping, especially for experiments containing a large number of samples, such as those involving recombinant inbred line populations [29]. In some phenotyping solutions for individual plants, the phenotyping platform has a fixed sensor and rotates the shoot on a turntable [54], which can cause leaves to shake on the turntable, thereby leading to data artifacts. In contrast, MVS-Pheno rotates the camera, thus leaving the shoot static. This approach reduces the noise caused by the slight movement of organs. However, a relatively larger space is needed compared with approaches that rotate shoots rather than cameras. Besides the automatic image acquisition hardware of this platform, MVS-Pheno also provides a matching data acquisition console, data processing and phenotype extraction software, and a data management system. This novel integration facilitates the application of the platform by users with various backgrounds, with a focus on agricultural users and plant breeder. Compared with the aforementioned MVS-based phenotyping solutions, MVS-Pheno is much more efficient and convenient to use and solves key practical issues.

### 5.3. Effects of Environmental Factors on the Platform

**Figure 9:**
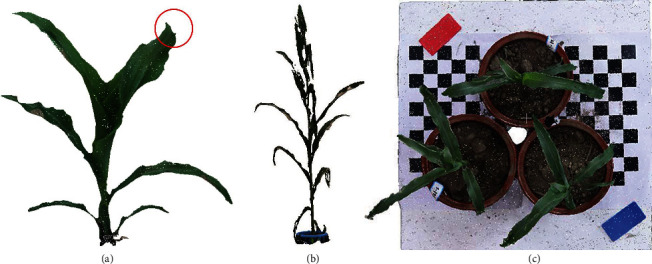
Illustration of environmental factors on MVS-Pheno. The points at the tip of the blade are lost when the wind speed is 2 m/s during data acquisition (a). Reconstructed shoot, with color distortion and point deficiency, using the images acquired under poor illumination (b). The use of black-and-white grid calibration plates benefits the quality of reconstructed point clouds.

The MVS-Pheno platform can be used beyond indoor environments, including in temporary tents near field sites or directly in fields under calm weather. In such cases, environmental conditions are essential factors that affect the usage of the MVS-Pheno platform. Herein, we discuss the influence of three major factors, including (1) wind, (2) illumination, and (3) ground texture. (1) Theoretically, 3D reconstruction requires the target plant to remain static during the data acquisition process. Two successive images captured under a gentle breeze exhibit differences in pixels captured, causing reconstruction errors, especially for the leaf blades. Experiments have demonstrated that the tips of blades cannot be reconstructed when the wind speed is greater than 1.6 m/s (Figure 9(a)). Therefore, the platform works best in the absence of wind. (2) Illumination is another key factor affecting the reconstruction accuracy of MVS. It is better to conduct image acquisition under natural light. Weak, strong, dim, or uneven lighting should be avoided. Poor illumination may lead to color distortion and/or deficiency of point clouds and even fail to generate point clouds (Figure 9(b)). Therefore, outdoor data acquisition should be conducted in cloudy weather when possible, and indoor acquisition should use flood lights if possible. (3) The ground texture also affects the accuracy of MVS reconstruction during data postprocessing. MVS reconstruction also includes the ground, and it requires that the background images have as rich a texture as possible. Therefore, rough, nonreflective, and textured ground may improve reconstruction accuracy. Trials have shown that chaotic backgrounds can actually produce better quality 3D reconstructions. In practice, the use of black-and-white grid calibration plates placed on the ground as a reference effectively improves the quality of reconstructed point clouds (Figure 9(c)).

### 5.4. Potential Application for Plant Phenotyping

Plants grow fast (i.e., a leaf emerges and expands about every three days at the early growth stage of maize), and long-term data acquisition staggered across different growth stages can create meaningless data for comparative analysis. Thus, effective data acquisition approaches are essential for high-throughput phenotyping. Because data acquisition of large-scale populations can be accomplished in a short time using MVS-Pheno, data can be acquired in a timely manner, with postprocessing conducted later. This data acquisition and postprocessing approach demands high-quality acquired data, and the homogeneity of multiview images around each shoot using MVS-Pheno is rather promising. Compared with expensive 3D scanners [35], MVS-Pheno is low-cost and thus feasible for use by most research teams.

Because the only physical limitation of the MVS-Pheno platform is that the shoot must fit within the device and have relatively few self-occlusions, it is apparently suitable for automatically acquiring image sequences for shoots of many other plant species, including wheat [55], soybean [38], and tobacco. Point clouds can be reconstructed using the acquired images. The only difference is that the empirical settings of the device parameters are distinct for various plants. For example, to obtain more morphological details of wheat shoots, more images at each layer are required. Regarding the phenotype extraction processes of the platform, however, the algorithms are not applicable to other plant species, as the software was custom-developed for maize according to its morphological characteristics.

The data management system is an important component of the MVS-Pheno platform. It facilitates data classification, storage, management, and usage. The database is scalable, and subdatabases can be customized for specific experiments. The data management system provides powerful support for further big data research on plant phenomics [56].

### 5.5. Future Improvements

The platform still needs to be improved to enable more convenient usage. Accordingly, the device will be developed to reduce its weight in order to facilitate transport and assembly. The postprocessing programs, in particular the skeleton extraction process for phenotype calculation, would benefit from optimization to improve its computational efficiency. Owing to the manual interfaces with and usage of third-party software, the postprocessing is segmented into several times. Accordingly, we aim to develop an integrated solution for individual plant phenotyping, with the ultimate objective of offering an online solution that realizes full automation, including data acquisition, processing, and analysis.

## Figures and Tables

**Table 1:** Item costs of the hardware device.

Items	Cost ($)
Main body of the device	4975
Two camera sensors	1700
Laptop	700
Wireless bar code scanner	70
Fittings	115
Total price	7560

^∗^The fittings include a portable power source, four LED white light sources, and cables for the device.

**Table 2:** The weight and minimum length parameters of the main components of the device.

Component	Identifier	Minimum length (cm)	Weight (kg)
Support table and rotary table	Part-A	90	65.8
Horizontal beams	Half of part-B	120	16.7
Upper part of the supporting arm	Half of part-C	120	8.4
Lower part of the supporting arm	Half of part-C	120	9.5
Supporting table for laptop	Part-D1	60	3.0
Balance weight	Part-D3	12	20.0

^∗^The lengths of the supporting arm and horizontal beams are adjustable. They are adjusted to their minimum lengths during transportation. Therefore, the minimum lengths are given rather than their full lengths.

**Table 3:** Standard empirical settings of the parameters for the console for maize shoot image acquisition.

Parameter	Representation	Empirical value	Unit
Total angle of rotation range	*φ*	400	°
Rotation velocity	*v*	6	°/s
Time interval for taking images	*t*	2	s
Number of images per layer	*n*	33	

**Table 4:** Morphological description of maize shoots at three growth stages.

Growth stage	Days after sowing	Averaged plant height (cm)	Fully expanded leaf number
V5	21	40.1	5
V15	51	180.0	15
R1	81	201.1	22

**Table 5:** Efficiency description of MVS-Pheno platform, including the time cost of data acquisition and processing and the corresponding parameter settings of the device for four growth periods of maize with different shoot sizes.

Sample ID	Sample description			Parameter settings of the device			Time cost (s)				
	GP	PHR (cm)	CO	CN	RoB (cm)	SRH (cm)	IAT	PCRT	SET	PET	TT
1	V6	40-60	No	1	50	60	60	403	140	2	605
2	V9	80-120	No	2	70	150	60	896	180	3	1139
3	V13	130-160	No	2	100	200	60	1060	220	3	1343
4	R1	190-250	No	3	150	300	60	1644	240	3	1947
5	R1	250-400	Yes	2	150	200	120	3288	300	10	3718

^∗^GP: growth period; PHR: plant height range; CO: cutoff; CN: camera number; RoB: radius of beam; SRH: supporting arm height; IAT: image acquisition time; PCRT: point cloud reconstruction time; SET: skeleton extraction time; PET: phenotype extraction time; TT: total time.

## Data Availability

The raw data of the images acquired using the MVS-Pheno platform and reconstructed point clouds are available upon request to the authors.

## Supplementary Materials

Supplementary Materials Supplementary material offers a compressed file that collects the device and separated components. The files are organized in corresponding parts with the format of “sldprt” and “sldasm.” These file formats can be visualized and edited using SolidWorks software. “jpg” format pictures of corresponding components are also provided to ensure the checking of the users without the SolidWorks.
