# Supplementary figures and images for: MVS-Pheno: A Portable and Low-Cost Phenotyping Platform for Maize Shoots Using Multiview Stereo 3D Reconstruction
Source: Plant Phenomics. 2020 Mar 12;2020:1848437. doi: 10.34133/2020/1848437 (PMC7706320; doi:10.34133/2020/1848437)

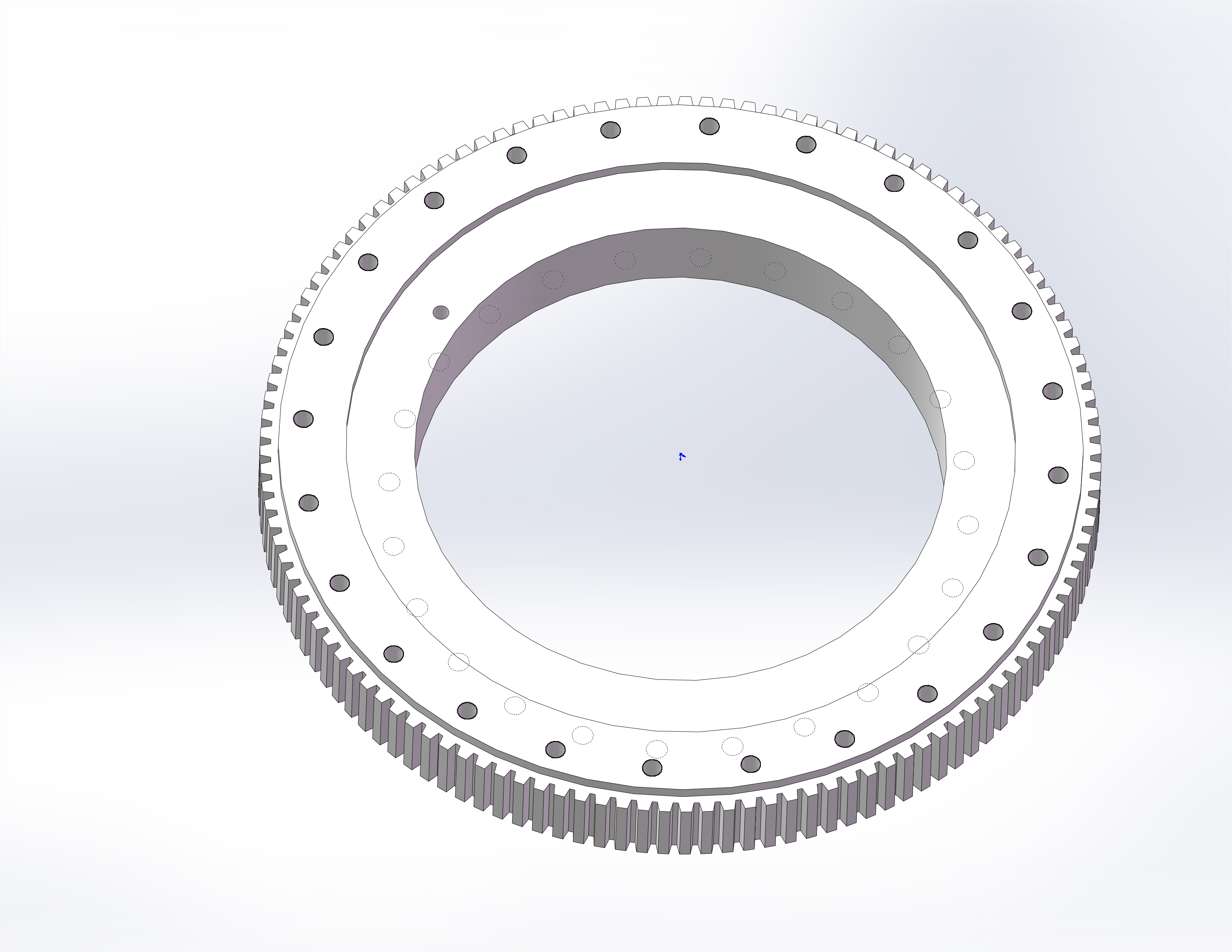

Supplement: Supplementary Materials — Supplementary material offers a compressed file that collects the device and separated components. The files are organized in corresponding parts with the format of “sldprt” and “sldasm.” These file formats can be visualized and edited using SolidWorks software. “jpg” format pictures of corresponding components are also provided to ensure the checking of the users without the SolidWorks. [file 1848437.f1.zip › A1.JPG]

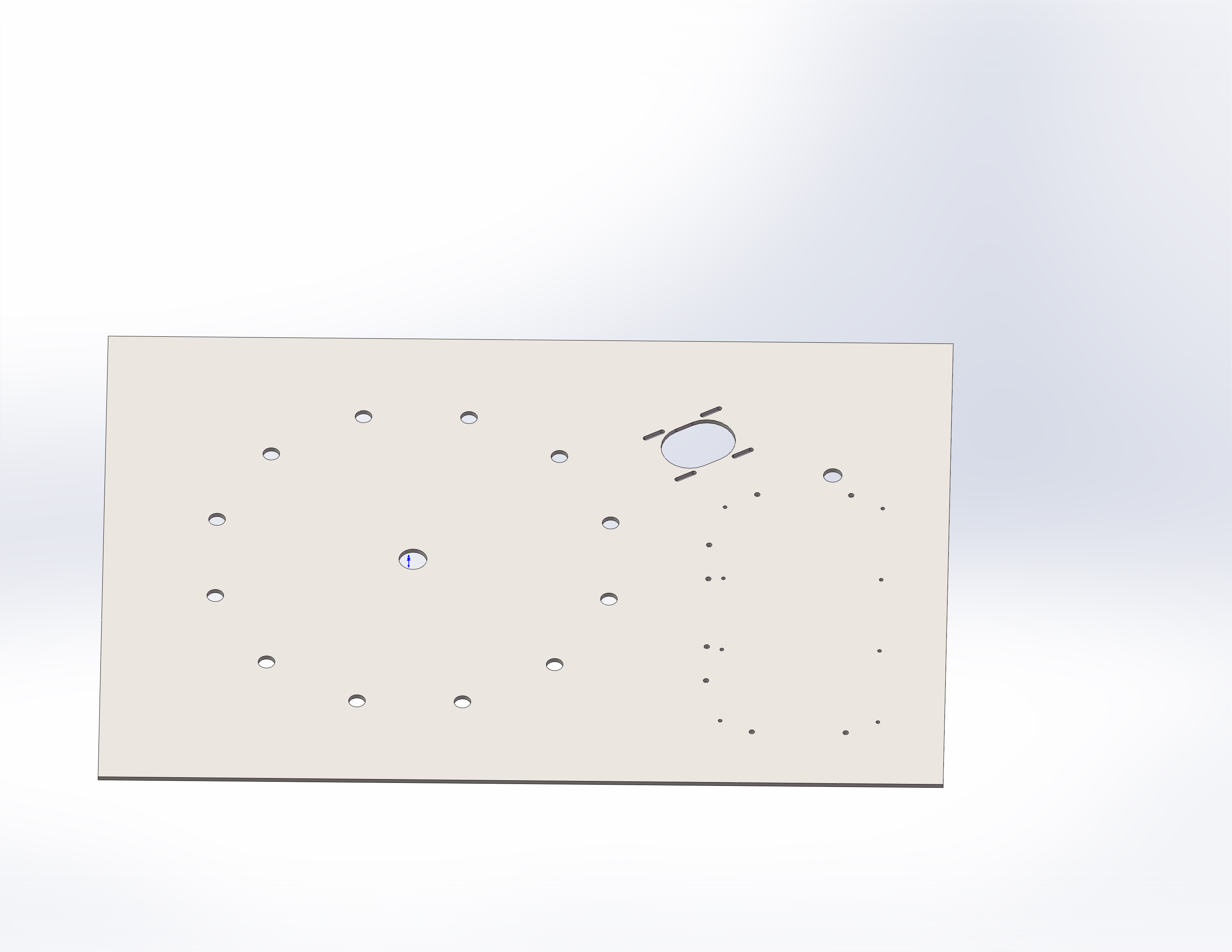

Supplement: Supplementary Materials — Supplementary material offers a compressed file that collects the device and separated components. The files are organized in corresponding parts with the format of “sldprt” and “sldasm.” These file formats can be visualized and edited using SolidWorks software. “jpg” format pictures of corresponding components are also provided to ensure the checking of the users without the SolidWorks. [file 1848437.f1.zip › A3-1.JPG]

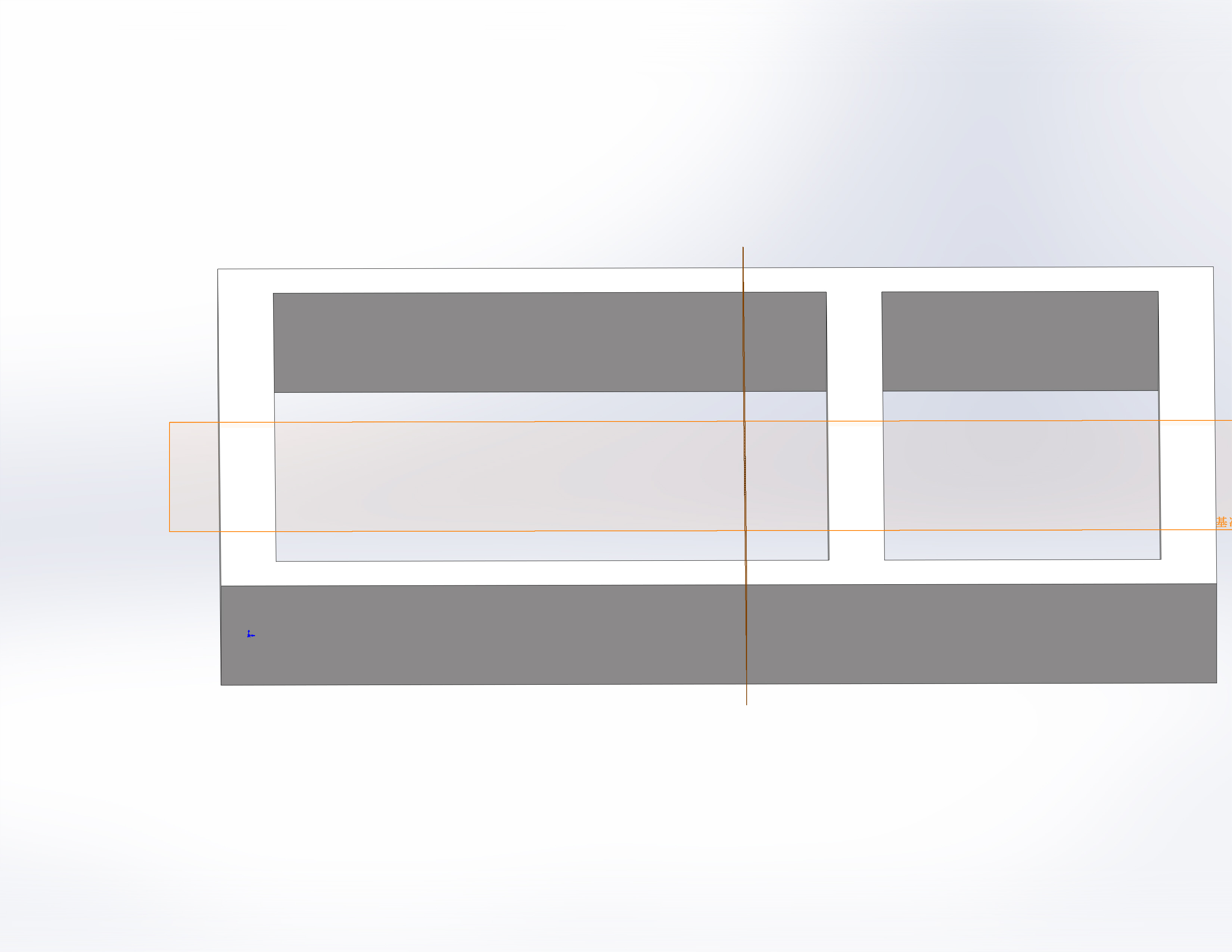

Supplement: Supplementary Materials — Supplementary material offers a compressed file that collects the device and separated components. The files are organized in corresponding parts with the format of “sldprt” and “sldasm.” These file formats can be visualized and edited using SolidWorks software. “jpg” format pictures of corresponding components are also provided to ensure the checking of the users without the SolidWorks. [file 1848437.f1.zip › A3-2.JPG]

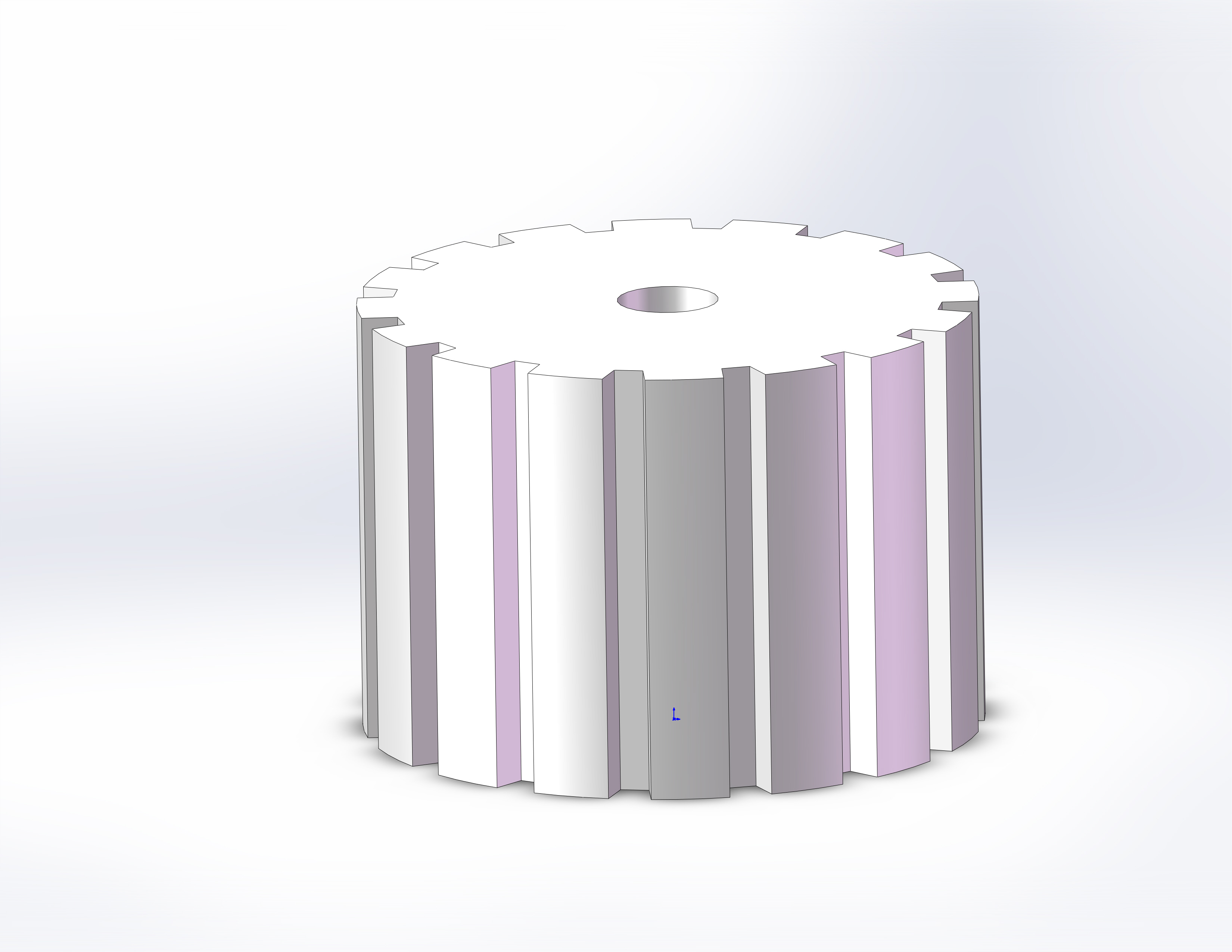

Supplement: Supplementary Materials — Supplementary material offers a compressed file that collects the device and separated components. The files are organized in corresponding parts with the format of “sldprt” and “sldasm.” These file formats can be visualized and edited using SolidWorks software. “jpg” format pictures of corresponding components are also provided to ensure the checking of the users without the SolidWorks. [file 1848437.f1.zip › A3-3.JPG]

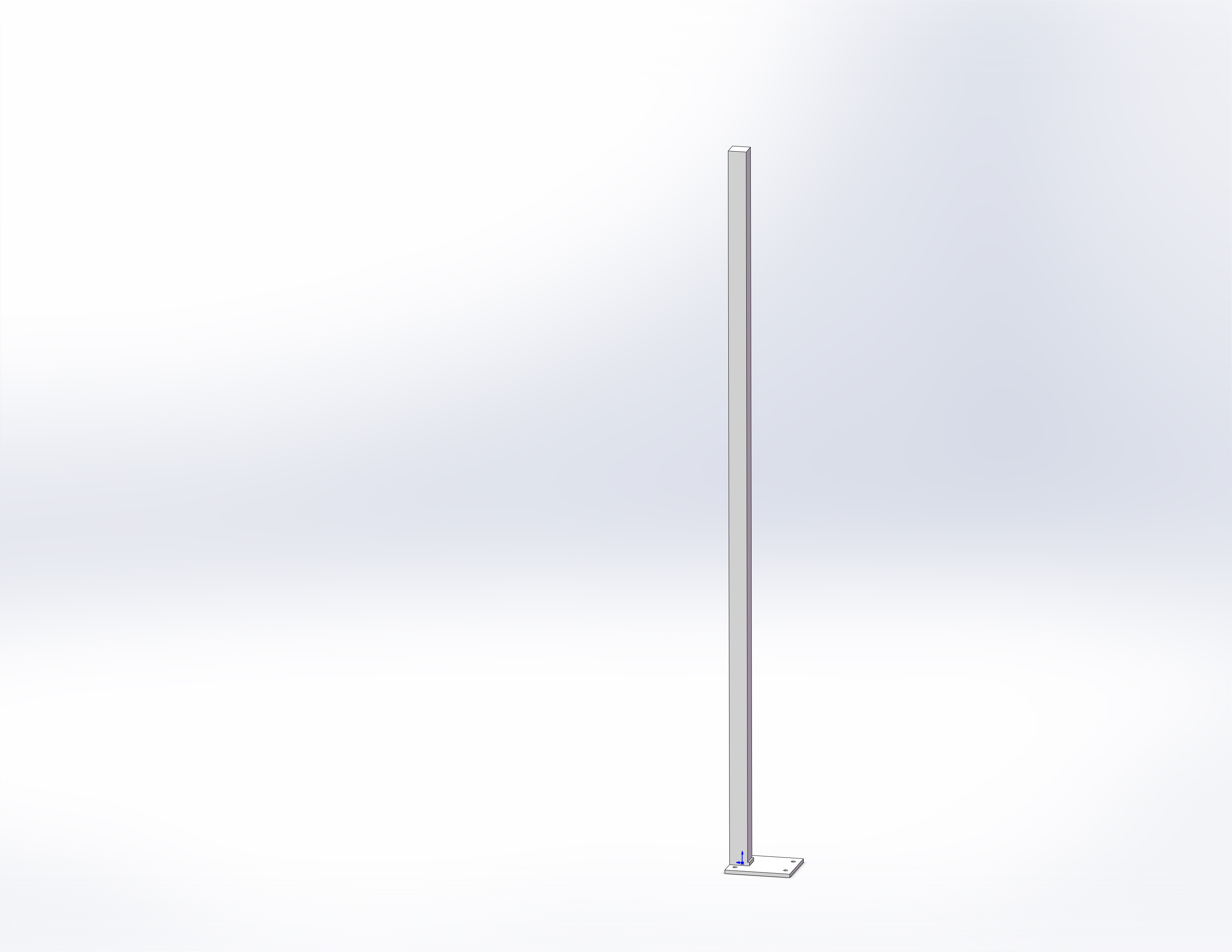

Supplement: Supplementary Materials — Supplementary material offers a compressed file that collects the device and separated components. The files are organized in corresponding parts with the format of “sldprt” and “sldasm.” These file formats can be visualized and edited using SolidWorks software. “jpg” format pictures of corresponding components are also provided to ensure the checking of the users without the SolidWorks. [file 1848437.f1.zip › C-1.JPG]

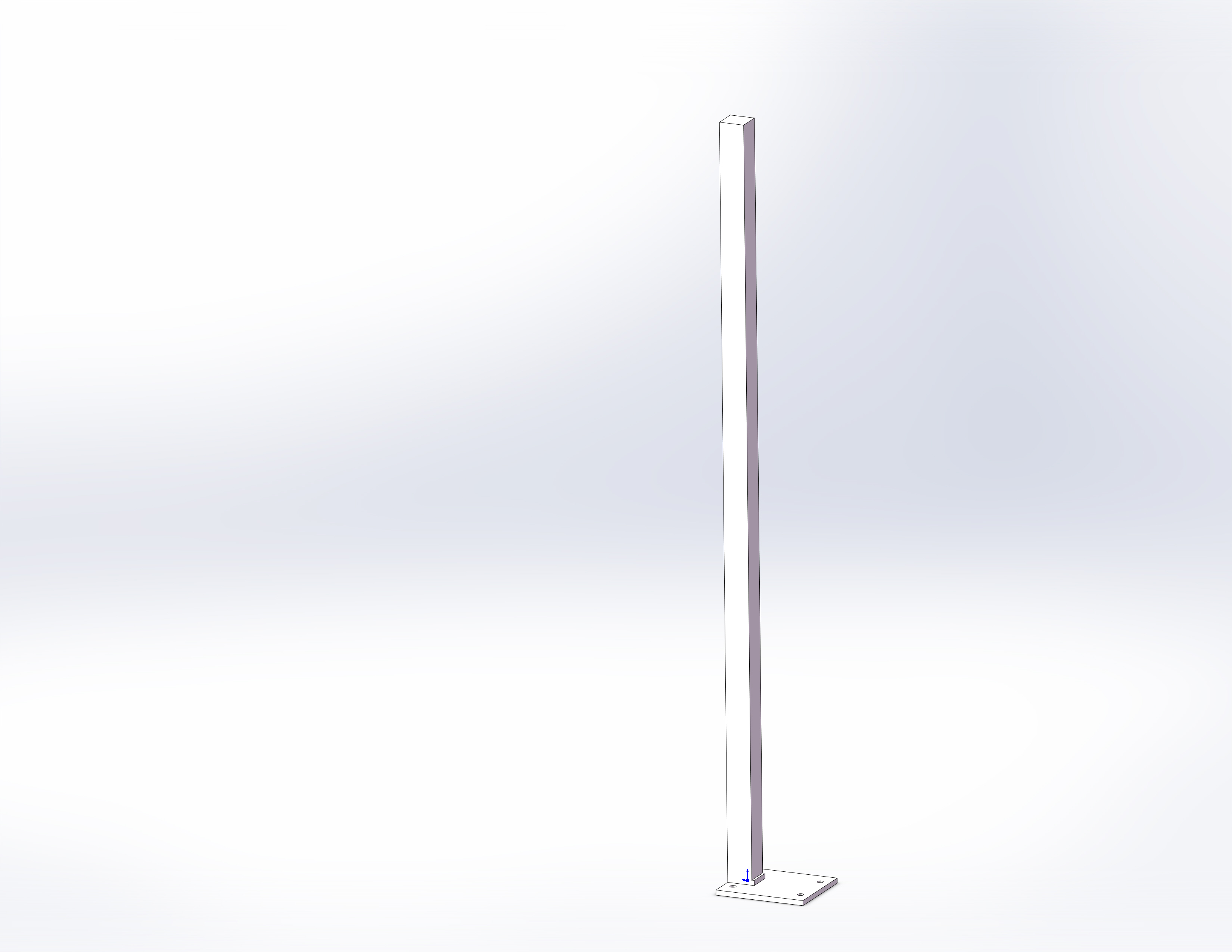

Supplement: Supplementary Materials — Supplementary material offers a compressed file that collects the device and separated components. The files are organized in corresponding parts with the format of “sldprt” and “sldasm.” These file formats can be visualized and edited using SolidWorks software. “jpg” format pictures of corresponding components are also provided to ensure the checking of the users without the SolidWorks. [file 1848437.f1.zip › C-2.JPG]

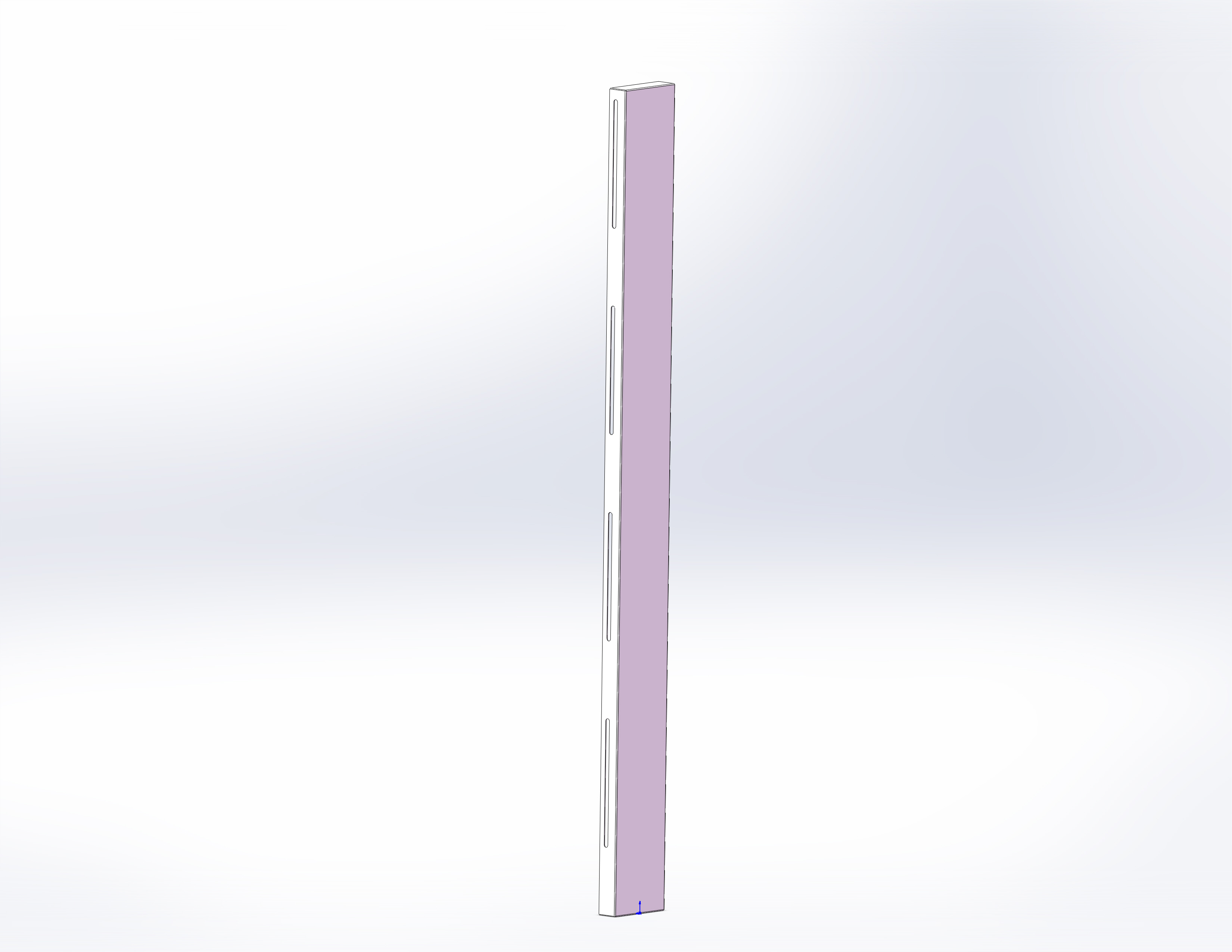

Supplement: Supplementary Materials — Supplementary material offers a compressed file that collects the device and separated components. The files are organized in corresponding parts with the format of “sldprt” and “sldasm.” These file formats can be visualized and edited using SolidWorks software. “jpg” format pictures of corresponding components are also provided to ensure the checking of the users without the SolidWorks. [file 1848437.f1.zip › C-3.JPG]

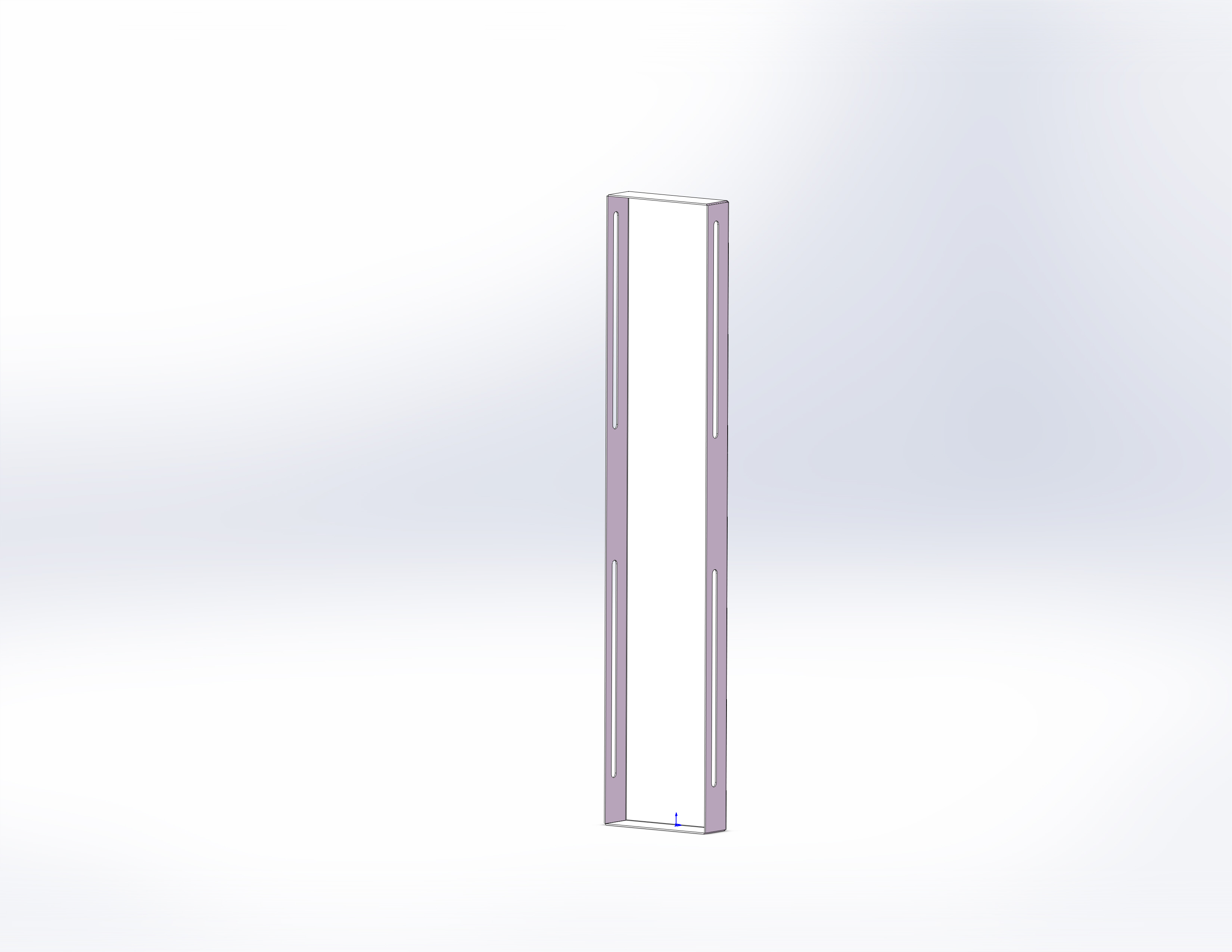

Supplement: Supplementary Materials — Supplementary material offers a compressed file that collects the device and separated components. The files are organized in corresponding parts with the format of “sldprt” and “sldasm.” These file formats can be visualized and edited using SolidWorks software. “jpg” format pictures of corresponding components are also provided to ensure the checking of the users without the SolidWorks. [file 1848437.f1.zip › C-4.JPG]

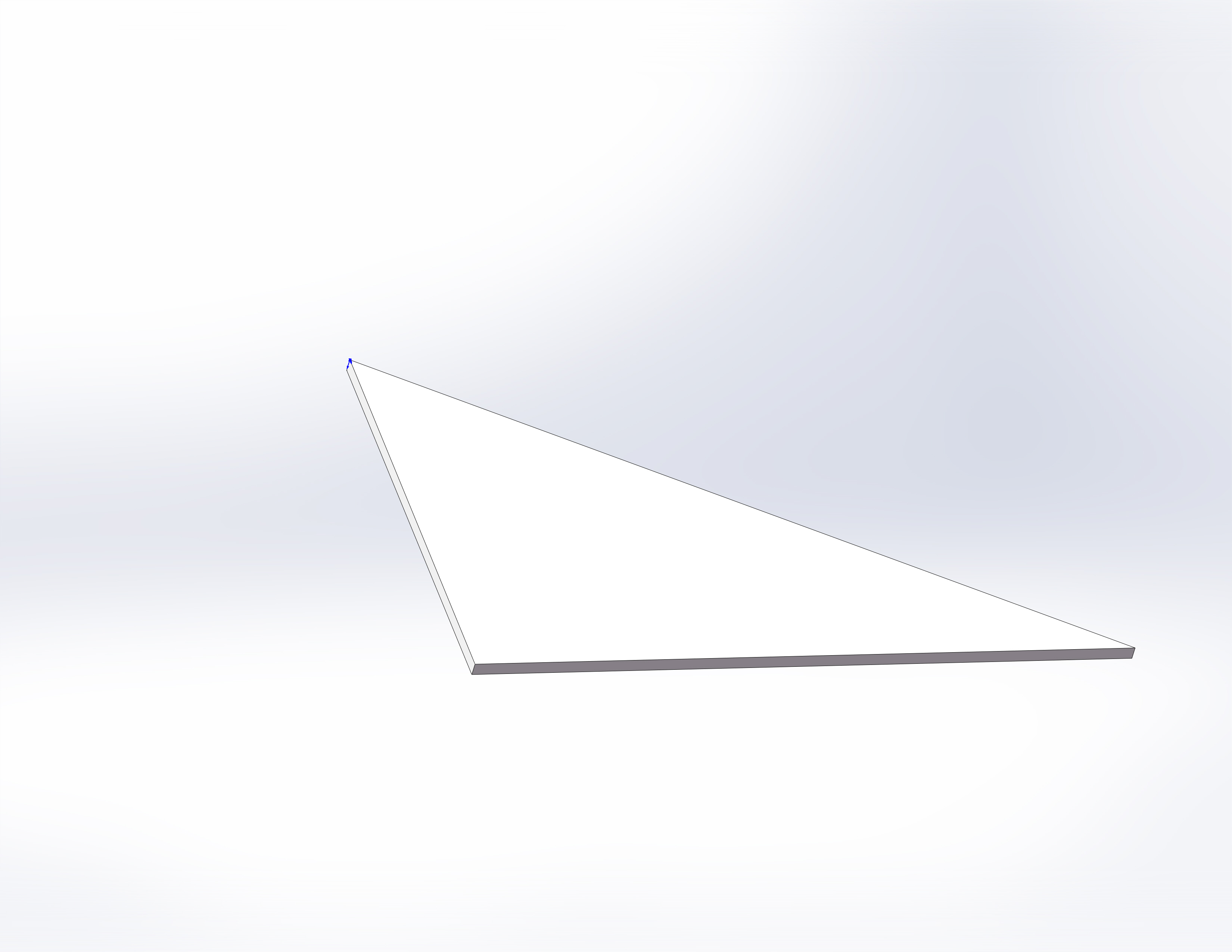

Supplement: Supplementary Materials — Supplementary material offers a compressed file that collects the device and separated components. The files are organized in corresponding parts with the format of “sldprt” and “sldasm.” These file formats can be visualized and edited using SolidWorks software. “jpg” format pictures of corresponding components are also provided to ensure the checking of the users without the SolidWorks. [file 1848437.f1.zip › C-5.JPG]

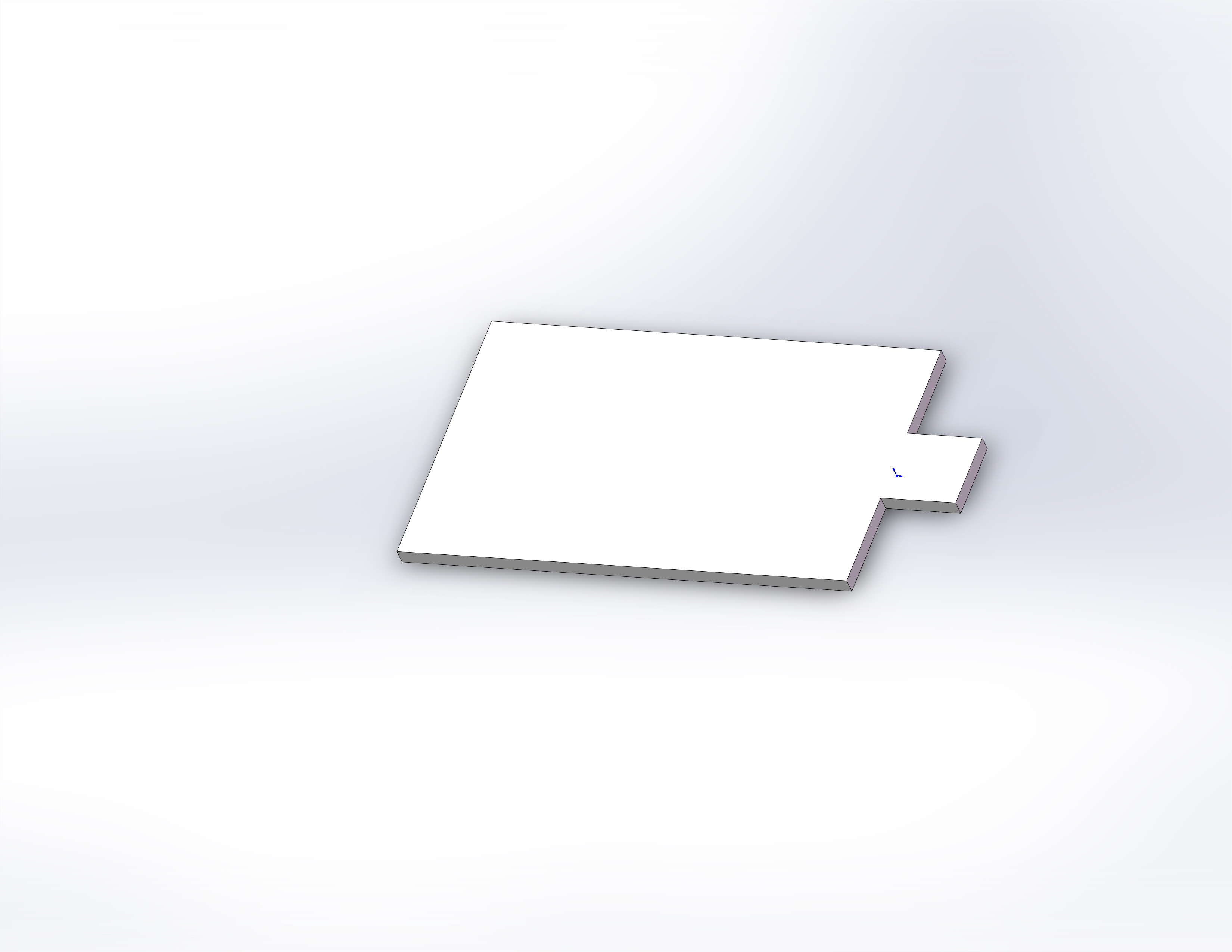

Supplement: Supplementary Materials — Supplementary material offers a compressed file that collects the device and separated components. The files are organized in corresponding parts with the format of “sldprt” and “sldasm.” These file formats can be visualized and edited using SolidWorks software. “jpg” format pictures of corresponding components are also provided to ensure the checking of the users without the SolidWorks. [file 1848437.f1.zip › C-6.JPG]

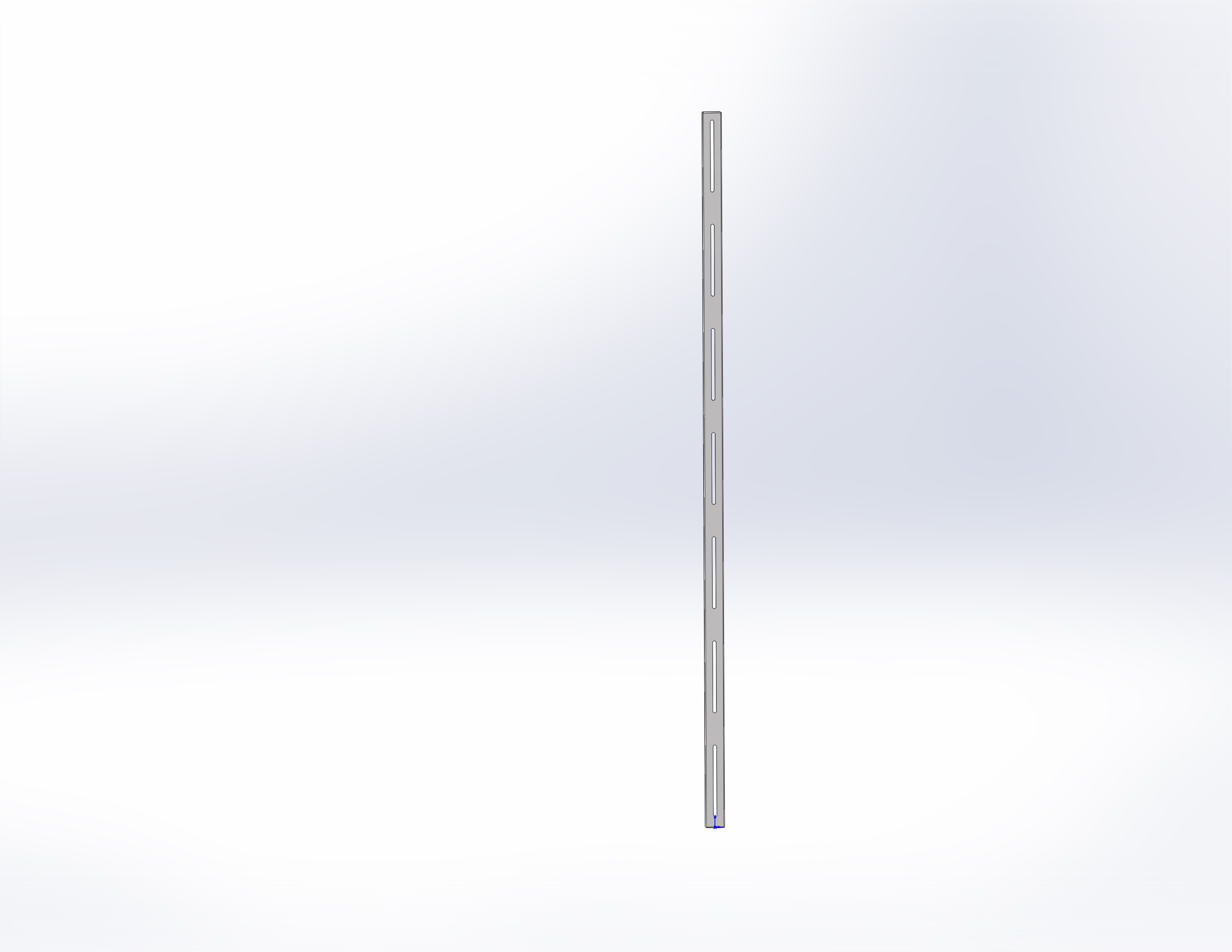

Supplement: Supplementary Materials — Supplementary material offers a compressed file that collects the device and separated components. The files are organized in corresponding parts with the format of “sldprt” and “sldasm.” These file formats can be visualized and edited using SolidWorks software. “jpg” format pictures of corresponding components are also provided to ensure the checking of the users without the SolidWorks. [file 1848437.f1.zip › C-7.JPG]

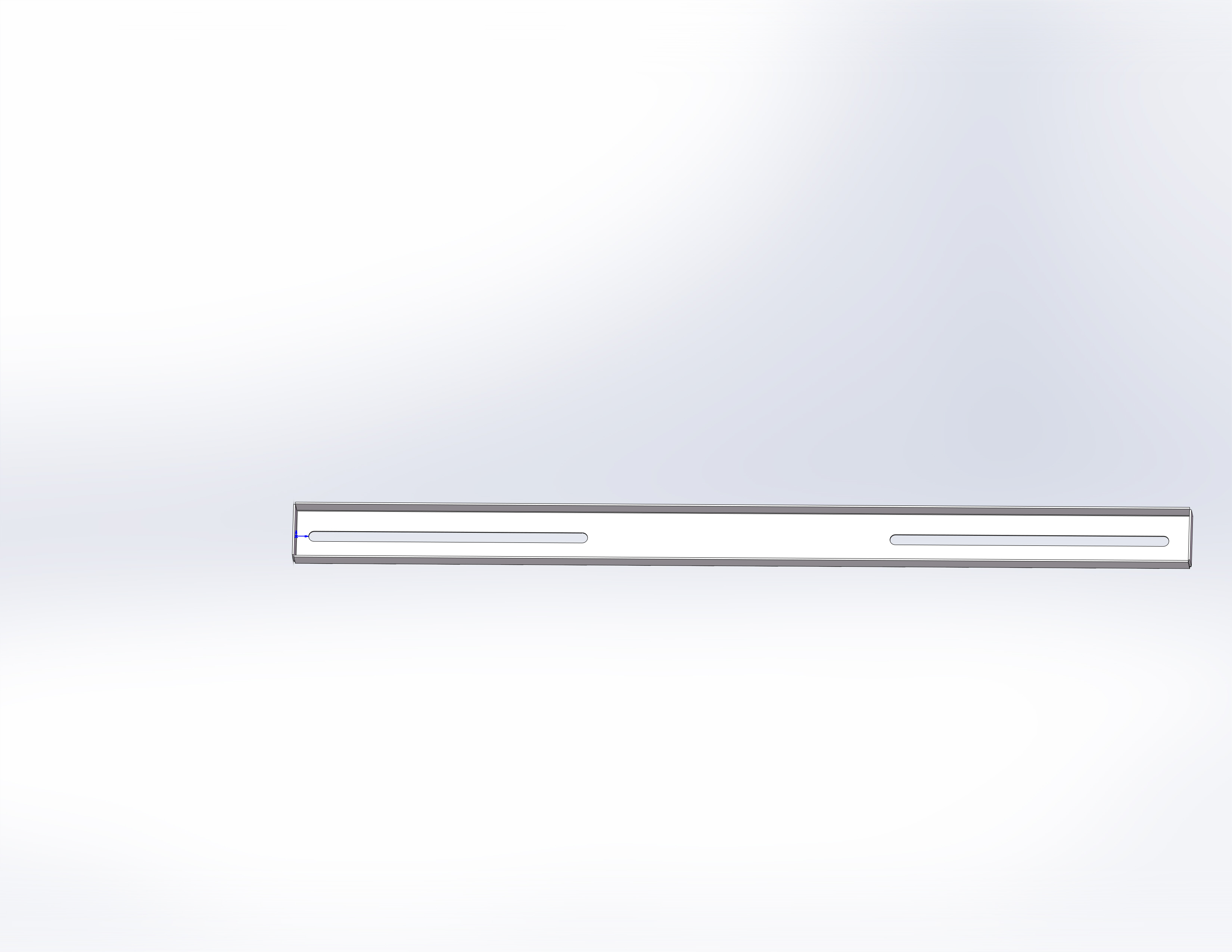

Supplement: Supplementary Materials — Supplementary material offers a compressed file that collects the device and separated components. The files are organized in corresponding parts with the format of “sldprt” and “sldasm.” These file formats can be visualized and edited using SolidWorks software. “jpg” format pictures of corresponding components are also provided to ensure the checking of the users without the SolidWorks. [file 1848437.f1.zip › C-8.JPG]

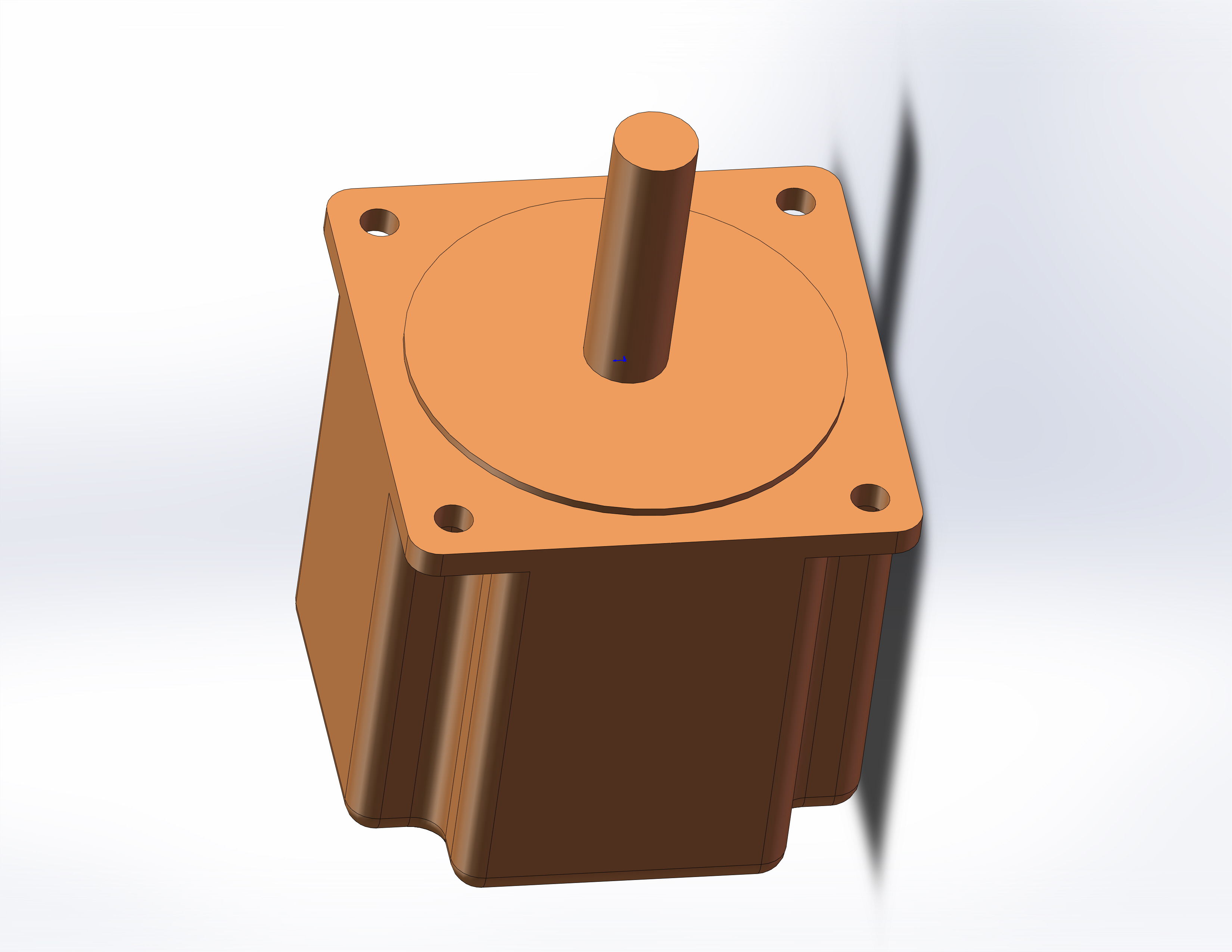

Supplement: Supplementary Materials — Supplementary material offers a compressed file that collects the device and separated components. The files are organized in corresponding parts with the format of “sldprt” and “sldasm.” These file formats can be visualized and edited using SolidWorks software. “jpg” format pictures of corresponding components are also provided to ensure the checking of the users without the SolidWorks. [file 1848437.f1.zip › Electric machinery inside the table.JPG]

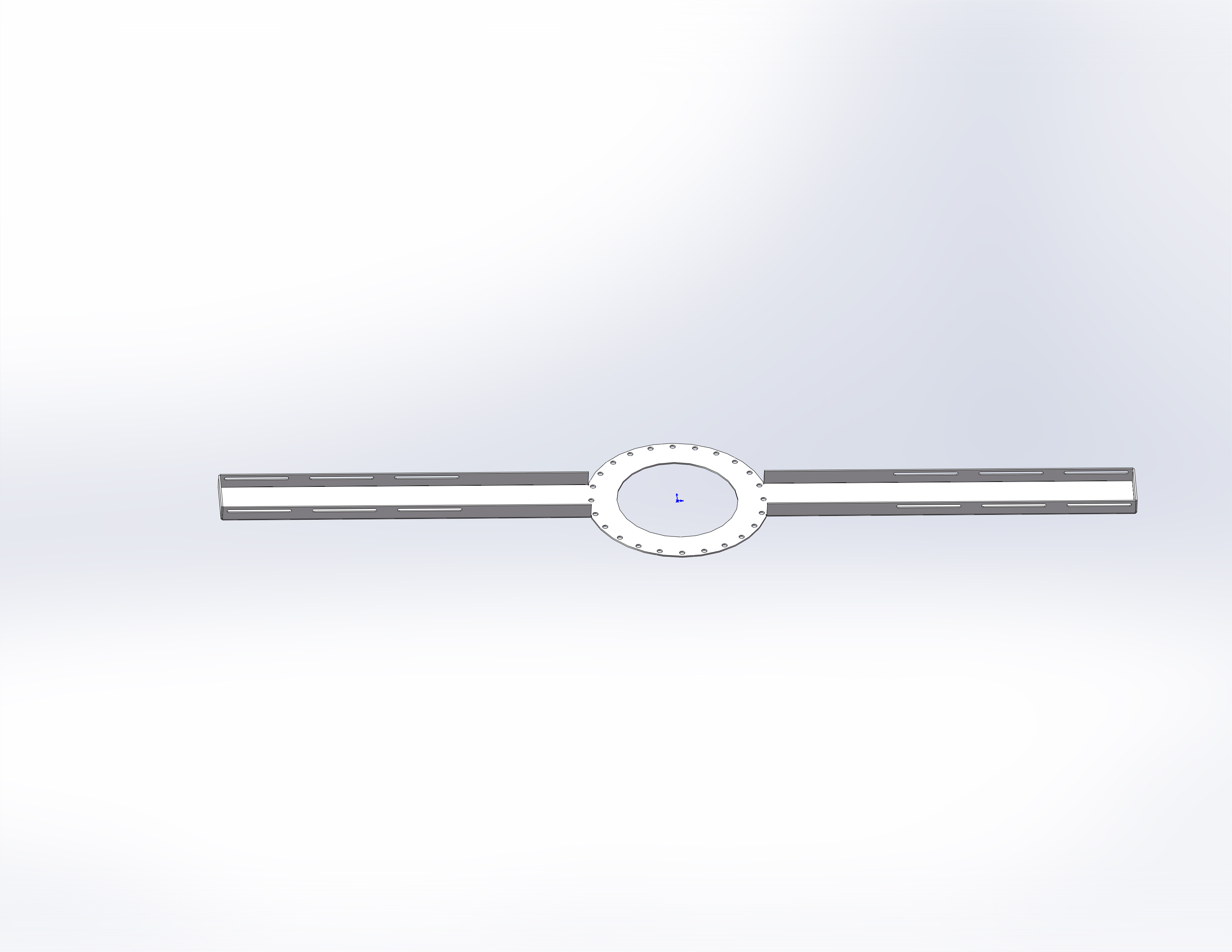

Supplement: Supplementary Materials — Supplementary material offers a compressed file that collects the device and separated components. The files are organized in corresponding parts with the format of “sldprt” and “sldasm.” These file formats can be visualized and edited using SolidWorks software. “jpg” format pictures of corresponding components are also provided to ensure the checking of the users without the SolidWorks. [file 1848437.f1.zip › Part-B.JPG]

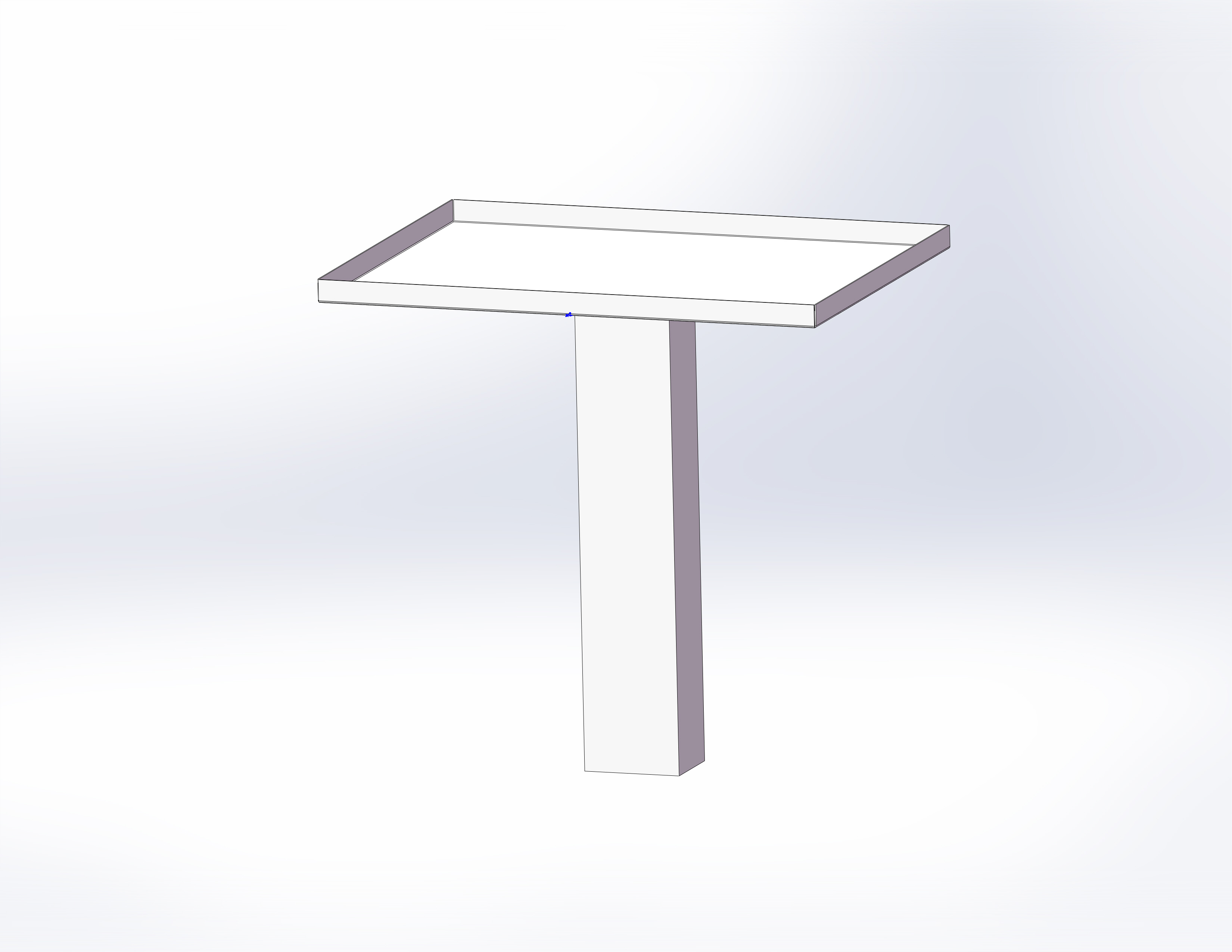

Supplement: Supplementary Materials — Supplementary material offers a compressed file that collects the device and separated components. The files are organized in corresponding parts with the format of “sldprt” and “sldasm.” These file formats can be visualized and edited using SolidWorks software. “jpg” format pictures of corresponding components are also provided to ensure the checking of the users without the SolidWorks. [file 1848437.f1.zip › Part-D.JPG]

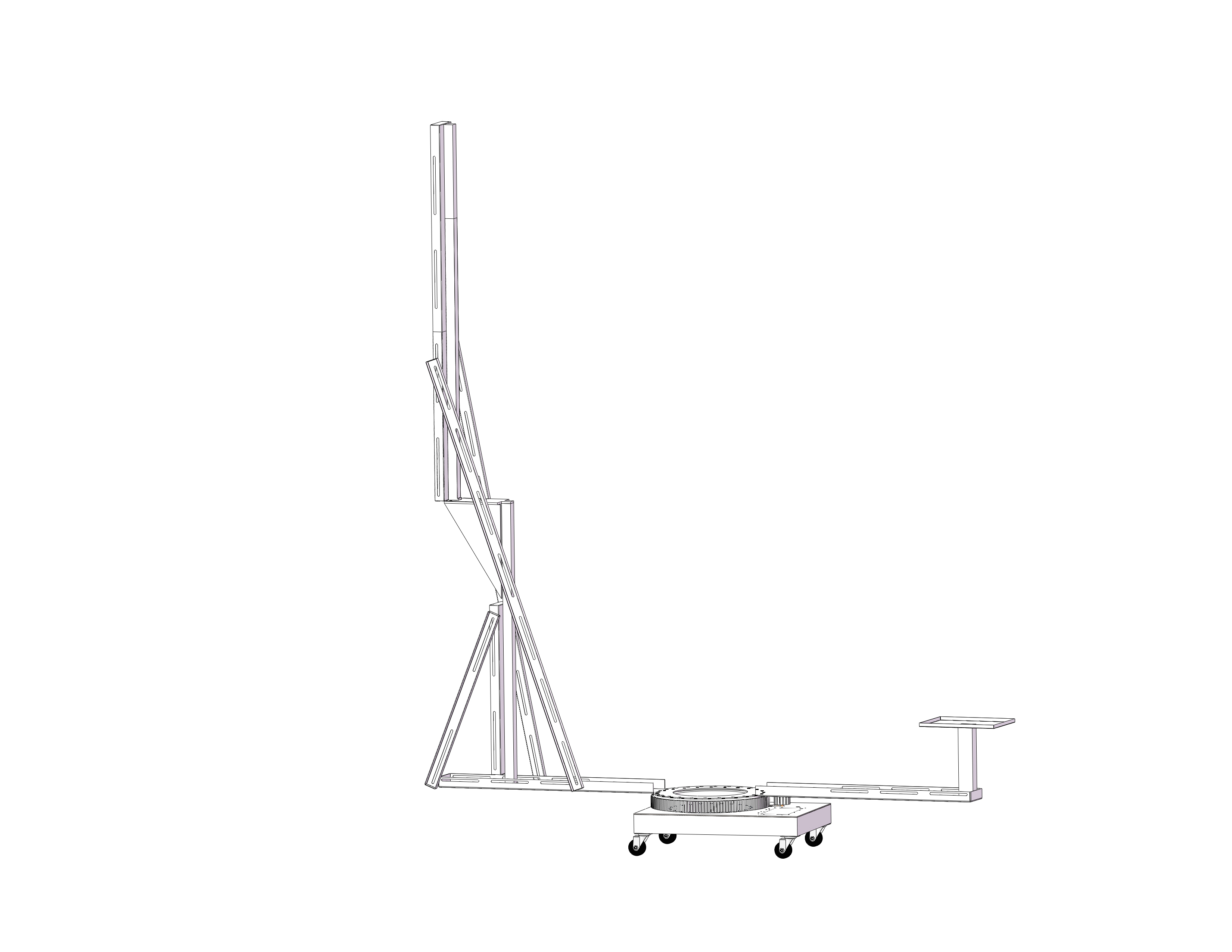

Supplement: Supplementary Materials — Supplementary material offers a compressed file that collects the device and separated components. The files are organized in corresponding parts with the format of “sldprt” and “sldasm.” These file formats can be visualized and edited using SolidWorks software. “jpg” format pictures of corresponding components are also provided to ensure the checking of the users without the SolidWorks. [file 1848437.f1.zip › The complete device.JPG]
